# High precision detection of conserved segments from synteny blocks

**DOI:** 10.1371/journal.pone.0180198

**Published:** 2017-07-03

**Authors:** Joseph MEX Lucas, Hugues Roest Crollius

**Affiliations:** IBENS, Département de Biologie, Ecole Normale Supérieure, CNRS, Inserm, PSL Research, University, Paris, France; Midwestern University, UNITED STATES

## Abstract

A conserved segment, i.e. a segment of chromosome unbroken during evolution, is an important operational concept in comparative genomics. Until now, algorithms that are designed to identify conserved segments often return synteny blocks that overlap, synteny blocks that include micro-rearrangements or synteny blocks erroneously short. Here we present definitions of conserved segments and synteny blocks independent of any heuristic method and we describe four new post-processing strategies to refine synteny blocks into accurate conserved segments. The first strategy identifies micro-rearrangements, the second strategy identifies mono-genic conserved segments, the third returns non-overlapping segments and the fourth repairs incorrect ruptures of synteny. All these refinements are implemented in a new version of PhylDiag that has been benchmarked against i-ADHoRe 3.0 and Cyntenator, based on a realistic simulated evolution and true simulated conserved segments.

## Introduction

Genomes are evolving molecules that are continuously mutating and rearranging. Despite these alterations, some segments of chromosomes remain exempt from disruption and still reflect the ancestral genome organisation; in 1984 they were first called “conserved segments” by Nadeau and Taylor [1]. Identifying those conserved segments is a prerequisite in rearrangement studies. However studies usually only focus on macro-rearrangements, to abstract themselves from spurious micro-rearrangements pervasive in draft genome assemblies, and thus rather use synteny blocks instead of conserved segments. In 2003, Pevzner and Tesler [2] introduced the term “synteny block” to refer to “segments that can be converted into conserved segments by micro-rearrangements. […] they usually consist of short regions of similarity that may be interrupted by dissimilar regions and gaps.” Studying synteny blocks and more generally identifying the conservation of synteny is the first step toward the identification of conserved segments from extant genomes. However considering that synteny blocks are a proxy for conserved segments is most of the time a mistake since numerous real micro-rearrangements, unrelated to genome assembly errors, are scattered in extant genomes [3,4]. Furthermore, because the identification of synteny blocks relies by definition on the conservation of synteny relationships between at least two markers, synteny blocks systematically miss conserved segments containing only one marker, and thus they cannot account for breakpoints corresponding to single-marker inversions. In this article we provide strategies to fine-tune the retrieval of conserved segments through the processing of synteny blocks.

**Fig 1:**
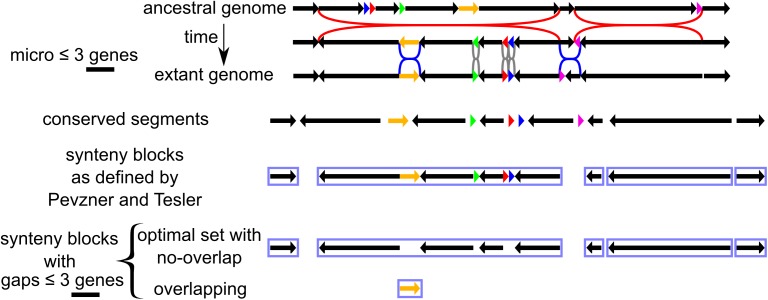
A scenario of rearrangements from one ancestral chromosome to one extant chromosome and corresponding conserved segments and synteny blocks. Along chromosomes, arrows represent uninterrupted segments of several genes and triangles represent segments of chromosomes composed of one gene. The three first lines describe the scenario of inversions, from the ancestral chromosome (first line) to the extant chromosome (3^rd^ line) in two steps: 2 macro-inversions and then 5 micro-inversions. Macro-inversions (reversing at least 4 genes) are in red whereas micro-inversions (reversing at most 3 genes) are in blue and grey: blue inversions reverse 3 genes and grey inversions reverse 1 gene. The two lines below show the corresponding conserved segments and “synteny blocks” (as intuitively defined first by Pevzner and Tesler [2]) while the last two lines show all synteny blocks corresponding to our formal definition. The set of optimal non-overlapping synteny blocks corresponds well to the original definition, except that the conserved segments nested in the gaps of our synteny blocks are not considered as part of the synteny blocks. In this example, the length of the maximum allowed gap in synteny blocks is the maximum length of micro-rearranged segments; *g* is equal to 3 genes.

More formally, we study genomes made of linear chromosomes with each chromosome being an ordered list of oriented genes (gene orientations are determined by their orientations of transcription into RNA). Only identifiable rearrangements are considered, i.e. breakpoints of rearrangements that change the position or orientation of at least one gene. In line with [1], we define a *conserved segment* from an initial ancestral genome to a set of descendant genomes as a maximum unbroken segment of ancestral genes. In other words it is a segment of ancestral genes that underwent no internal breakpoint, no internal disruption of ancestral gene order and no disruption of ancestral gene orientations, in all lineages from the initial genome to the set of descendant genomes of interest. An *ancestral gene* is a gene present in the ancestral genome and it may have been conserved in one or several extant genomes. After the ancestor, a gene that originated due to a *de novo* gene birth or to a duplication, is not an ancestral gene and a rearrangement that disrupted only non-ancestral genes does not change conserved segments. The exhaustive set of conserved segments is thus the set of fragments of the ancestral genome after (i) splitting at all rearrangement breakpoints involving ancestral genes that occurred between the ancestral genome and the descendant genomes and after (ii) removing all ancestral genes that have been deleted during evolution in at least one of the considered lineages. As for a conserved segment, a *synteny block* with gaps ≤ *g* is a maximum cluster of syntenic and neighbouring ancestral genes (at least two) whose gene order and gene orientations have been conserved during evolution. *g* is equal to the upper limit of ancestral genes within the gaps of synteny blocks, and neighbouring ancestral genes of a synteny block are spaced by a gap of at most *g* ancestral genes that do not belong to the synteny block. Contrary to a conserved segment, micro-rearrangements (rearrangements of segments of at most *g* ancestral genes) may have occurred between the neighbouring ancestral genes of a synteny block. It follows from these definitions that (i) there is no difference between a synteny block with no gap (*g* = 0) and a conserved segment (ii) a synteny block with *n* non-null gaps is made of *n+1* conserved segments spaced by these *n* non-null gaps. Except for the variation of ancestral gene content, a synteny block can be converted into a segment of the ancestral genome if, within its gaps, all the small conserved segments are re-ordered and reoriented by reversing the micro-rearrangements that generated them (natural micro-rearrangements, mainly small inversions, during evolution plus artificial micro-rearrangements due to annotation errors or assembly errors). All the micro-rearrangements here are identical to the “micro-rearrangements” in the intuitive definition of Pevzner and Tesler cited above. Fig 1 illustrates these definitions by considering the evolution of a chromosome rearranged by inversions along with all its corresponding conserved segments and synteny blocks. Another evolution could involve several lineages where genomes are made of several chromosomes rearranged by other types of rearrangements (reciprocal translocations, fissions, fusions transpositions,…) and edited by genic events (gene duplications, gene deletions and *de novo* gene births), the deduction of the corresponding conserved segments and synteny blocks would follow as well from the definitions. S1, S2 and S3 Figs depict more detailed evolutions of conserved segments corresponding to the evolution of an ancestral genome to one (S2 Fig) or two descendants (S3 Fig) with genic events and several types of rearrangements.

In this work we present five general issues that affect the identification of conserved segments: duplications of ancestral genes, micro-rearrangements, mono-genic conserved segments, overlaps of portions of diagonals and ambiguous conservation of gene order. We also introduce five strategies to solve them.

All strategies are implemented in a new version of PhylDiag [5], which we benchmarked against simulated conserved segments. To our knowledge there is no program that specifically identifies conserved segments as opposed to synteny blocks. We thus compared PhylDiag with i-ADHoRe 3.0 [6] and Cyntenator [7], two well-known alternative approaches to identify synteny blocks [8], and, during the comparison, we used their synteny blocks (more precisely base_clusters and *Representative Synteny Blocks*, RSBs) as if they were conserved segments. Finally, we explain false positive and false negative extremities that remain after our post-processing steps.

## Materials and methods

### Definitions and model

Inputs of all algorithms are genomes and gene families. A genome is usually a set of linear chromosomes and a chromosome is an ordered list of oriented genes. The orientation of each chromosome is arbitrarily chosen and either ranks genes in one order or the reverse; once the order is fixed, a chromosome has a first gene and a last gene. Each gene of a chromosome has an *orientation*: if the transcriptional orientation of the gene (5’ to 3’) points toward the last gene of the chromosome, its orientation is positive (+1); otherwise its orientation is negative (-1).

Genomes are altered by events during evolution and, in the field of vertebrate genome evolution, the most cited events altering genomes are of two types: genic events (gene duplications, either tandem or dispersed, gene deletions and *de novo* gene births) and chromosomal rearrangements (inversions, reciprocal translocations, fissions and fusions) (S14 Fig).

A gene evolves through duplication and speciation events and all its descendants form a family. A gene family is thus a set of genes that derive from one common ancestral gene. Pairs of genes of the same family are called homologs and they correspond to a homology relationship. The *matrix of homologies* of the comparison of two genomes is a sparse matrix where each row corresponds to a gene of the first genome and each column corresponds to a gene of the second genome; genes are ordered along chromosomes and chromosomes are sorted by decreasing chromosome lengths. This matrix can be represented as an array of *signs* equal to +, − or 0. Non-0 signs correspond to *homology signs*: a + sign means that the two corresponding homologs (genes of the same family corresponding to the row and the column of the homology) have the same orientation (+1 and +1; or -1 and -1) and a—sign means that they have reversed orientations (+1 and -1; or -1 and +1); S4 Fig gives a graphical example of a matrix of homologies of two genomes. If the first genome has n1 chromosomes and if the second genome has n2 chromosomes, this matrix is composed of n1 x n2 sub-matrices of homologies of the comparison of pairs of chromosomes.

In this work we study the conservation of synteny blocks and conserved segments from one ancestor to a pair of descendant species. We explained in [5] and in more details in [9] that a synteny block conserved along both lineages, with gaps ≤ *g* takes the shape of a consistent diagonal with gaps ≤ *g* in the homology matrix of compared extant genomes, as long as they are both *perfectly filtered* in such way that they contain only ancestral genes, conserved from the ancestor to both extant genomes.

A *consistent diagonal* (subsequently referred to as diagonal) is either a bottom-left to top-right diagonal with + signs (homologous genes that have the same respective orientations), or a top-left to bottom-right diagonal with–signs (genes in opposite orientations). In a diagonal, the gap between two successive homologies is computed using the Chebyshev distance metric and is thus equal to the maximum of the gaps between corresponding homologs in compared genomes. A conserved segment (which is a synteny block with *g* = 0) takes the shape of a consistent diagonal with no gaps when both compared genomes are perfectly filtered (S5 Fig). We will compare pairs of sequenced genomes to identify synteny blocks and conserved segments through the detection of these diagonals. A comparison only gives information on what happened after the most recent common ancestral genome (MRCA) of compared genomes, thus we will focus on the evolution from the MRCA to both compared genomes.

If gene trees of compared genomes are available, we define families from pruned phylogenetic gene trees [5]. A phylogenetic gene tree is a binary gene tree that represents the evolution of one initial gene and its later copies. The initial gene is at the root of the gene tree, nodes of the tree correspond to duplication events or speciation events and leaves correspond to genes, in extant genomes, deriving from the initial gene.

Given a pair of compared genomes, and corresponding gene trees, we prune gene trees at the level of the MRCA. After pruning, each root of a gene tree is an ancestral gene of the MRCA (we discard gene trees deriving from *de novo* gene births posterior to the MRCA). Each family is then defined as a set of genes in the same pruned gene tree. As a consequence, two genes are homologs (are in the same family) if and only if they derive from the same ancestral gene of the MRCA. With error-free gene trees, the pruning process differentiates gene lineages arising from paralogs in the MRCA, especially lineages of paralogs in the same cluster of tandem duplicates (S8 Fig).

See [5] and [9] for more formal definitions of genomes, gene orientations, homology relationships, matrices of homologies, signs of homologies, diagonals, distance metrics, gaps, gene trees, pruning of gene trees and families used in this article.

Using gene families as defined above, we pre-process compared extant genomes to get them as close as possible to the perfectly filtered genomes, that would only contain ancestral genes conserved in both species (S4, S5 and S6 Figs). As in other methods, we remove genes with no homolog in the other genome although this keeps unwanted copies of ancestral genes in genomes (S6 Fig). Contrary to diagonals when genomes are perfectly filtered (S5 Fig), these non-ancestral genes create *artificial gaps* in diagonals of conserved segments (if they come from dispersed duplications) or packs of homologies (if they come from tandem duplications) that disrupt the linearity of diagonals (S6 Fig). Errors in sequences and errors in annotations of compared genomes will cause additional artificial gaps.

Experience shows that artificial gaps are frequent in data. As a consequence, conserved segments take the shape of diagonals with some non-null gaps (S9 and S10 Figs). In addition, because of these artefacts, the maximum gap, g, of the definition of synteny blocks may thus differ from the maximum gap of corresponding diagonals, gapMax. In other words, synteny blocks with gaps ≤ g may take the shape of diagonals with some gaps > g; i.e. diagonals with gaps ≤ gapMax where gapMax is an integer strictly higher than g.

## Results

### Five issues and corresponding strategies for the detection of synteny blocks and conserved segments

**Fig 2:**
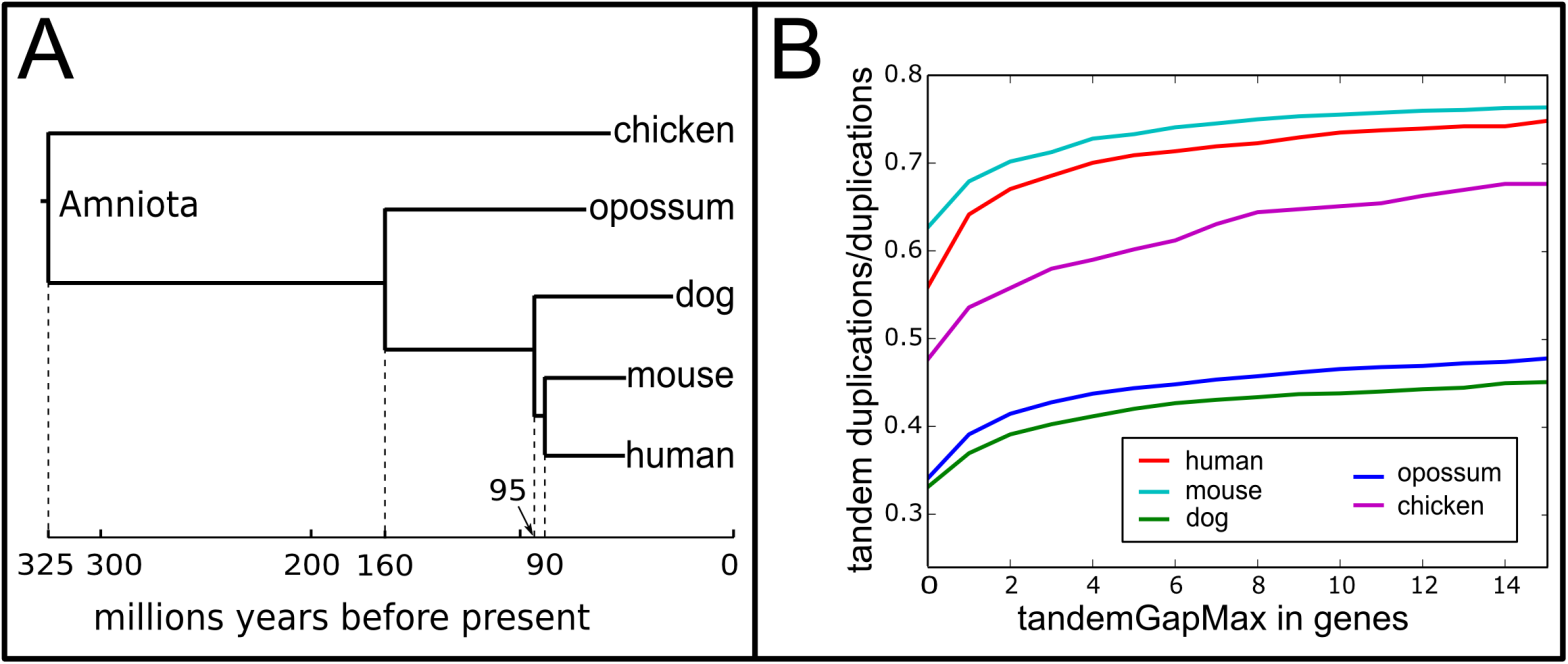
Proportion of the number of tandem duplications among all gene duplications that occurred between the Amniota ancestor and five extant vertebrates. Panel **A** contains the species tree linking extant human, mouse, dog, opossum and chicken species to Amniota, their most recent common ancestor. The topology of the tree and the dates of speciation come from the Ensembl database [11]. The graph in Panel **B** shows the proportion of tandem duplications among all duplications. Genes are considered duplicated in tandem if they fulfil two criteria: (i) they must belong to the same gene family, and therefore share the same ancestral Amniota gene (ii) they are separated, in the extant genome, by at most *tandemGapMax* genes (on the x-axis). Tandemly duplicated genes form clusters of two or more tandem duplicates of the same gene family. The number of tandem duplication events within a cluster is estimated as the number of tandem duplicates minus 1 (the original ancestral gene). Computations are performed using genomes from Ensembl v81 and the corresponding gene trees of Ensembl Compara. The proportion of tandem duplications among all duplications is substantial and varies from approximately 40% to 70% depending on the lineage.

The first issue for the detection of synteny blocks and conserved segments is caused by numerous tandem duplications [10] (S6 Fig) in genomes of vertebrates (Fig 2).

A tandem duplication in one lineage adds a new non-ancestral gene that should be filtered out. Unfortunately removing non-ancestral genes from extant genomes is often impossible, especially within clusters of tandem duplicates where the ancestral gene and its surrounding tandem duplicates cannot be distinguished. To bypass this difficulty, it has previously been suggested to collapse each cluster of tandem duplicates to a unique gene [12,13] that is then considered as representing the ancestral gene (S8 Fig). Here we explain a generalisation of this pre-processing method and show some of its limits based on real data. We also explain why dispersed duplications remain an issue that forces us to allow gaps within diagonals corresponding to conserved segments.

The second issue concerns the identification of “micro-rearrangements”. It was a key element in the debate between the fragile breakage model and the random breakage model; and the associated debate about the real rate of breakpoints reuse [14,15]. For example, Pevzner and Tesler discarded “micro-rearrangements” smaller than 1Mb with GRIMM to circumvent errors in genomic sequence assemblies but at the cost of rejecting genuine micro-conserved segments and simultaneously suppressing internal breakpoints within their blocks [2].

The third issue involves conserved segments of one gene. According to our definition such a segment corresponds to one ancestral gene flanked by breakpoints on both sides. In previous studies, either synteny blocks of at least two genes are studied or mono-genic inversions are studied [3], but we do not know any case where both are studied together whereas all conserved segments, whatever their lengths, are interesting for rearrangement studies.

The fourth issue concerns solving “conflicts” between diagonals of putative synteny blocks [16]. Overlaps of diagonals, often referred as overlaps of synteny blocks [16], must be removed for genome rearrangement studies [17,18] that mainly use non-overlapping synteny blocks as a basis to define the rearrangement scenario that transforms one genome into another. Except in a few cases [19,20], algorithms do not eliminate overlaps [16].

Finally, the fifth issue concerns synteny blocks erroneously shortened while solving conflicts between diagonals, due to local ambiguities in the conservation of the ancestral gene order.

While the first issue is tackled by collapsing tandem duplicate clusters, here we also describe refinement steps to resolve the four remaining issues. The first step identifies mono-genic conserved segments. The second step identifies micro-rearrangements and corresponding breakpoints. The third refinement step solves overlaps and yields an optimised set of non-overlapping conserved segments. Finally, the last step truncates diagonals and merges previously overlapping diagonals to recover complete synteny blocks from erroneously shortened blocks.

### Pre-processing

#### Collapsing clusters of tandem duplicates

**Fig 3:**
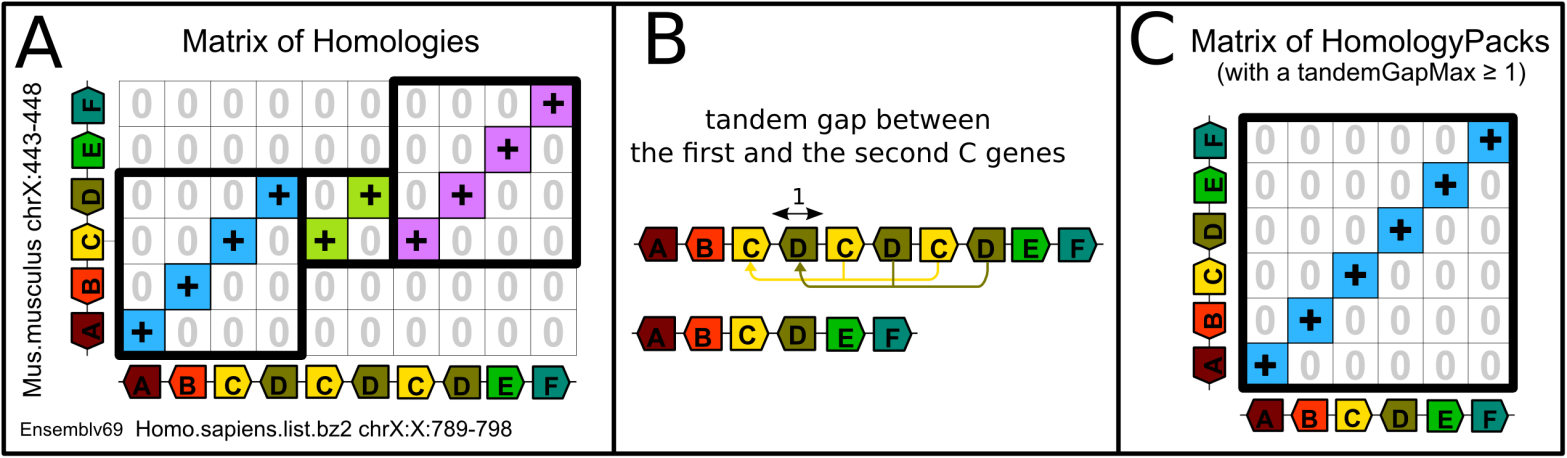
Collapsing a cluster of tandem duplicates can circumvent ruptures of collinearity caused by tandem segmental duplications. Panel **A** contains the matrix of homologies of the comparison of a segment of the human chromosome X and a segment of the mouse chromosome X from Ensembl v69. Two segmental tandem duplications of human genes C and D blur the conservation of the ancestral gene order. Panel **B** details the process of collapsing the clusters of tandem duplicates on the human segment. If *tandemGapMax* ≥1 there are two clusters, a cluster of 3 genes of family C and a cluster of 3 genes of family D. The three C genes form a cluster because less than *tandemGapMax* other genes separate any pair of C genes. All genes in a cluster are collapsed at the location of the first gene, as indicated by the yellow arrow. The same applies with the cluster of the D family. When tandem duplicates are collapsed, the conserved order of ancestral genes now forms an uninterrupted linear diagonal in the matrix of homology packs [5] in Panel **C**. Bounding boxes are drawn as black rectangles around diagonals in panel A and around the diagonal of the identified conserved segment in panel C.

This pre-processing step rewrites chromosomes and returns a unique representative location and a unique representative orientation per cluster of tandem duplicates. Two genes are clustered if they are separated by at most *tandemGapMax* genes, which is a user-defined parameter. Then a unique location is chosen for the representative locus; usually the index of the first gene of the cluster, and the chromosome is rewritten (Fig 3B). This method circumvents interruptions of collinearity caused by segmental tandem duplications of lengths ≤ *tandemGapMax*.

**Fig 4:**
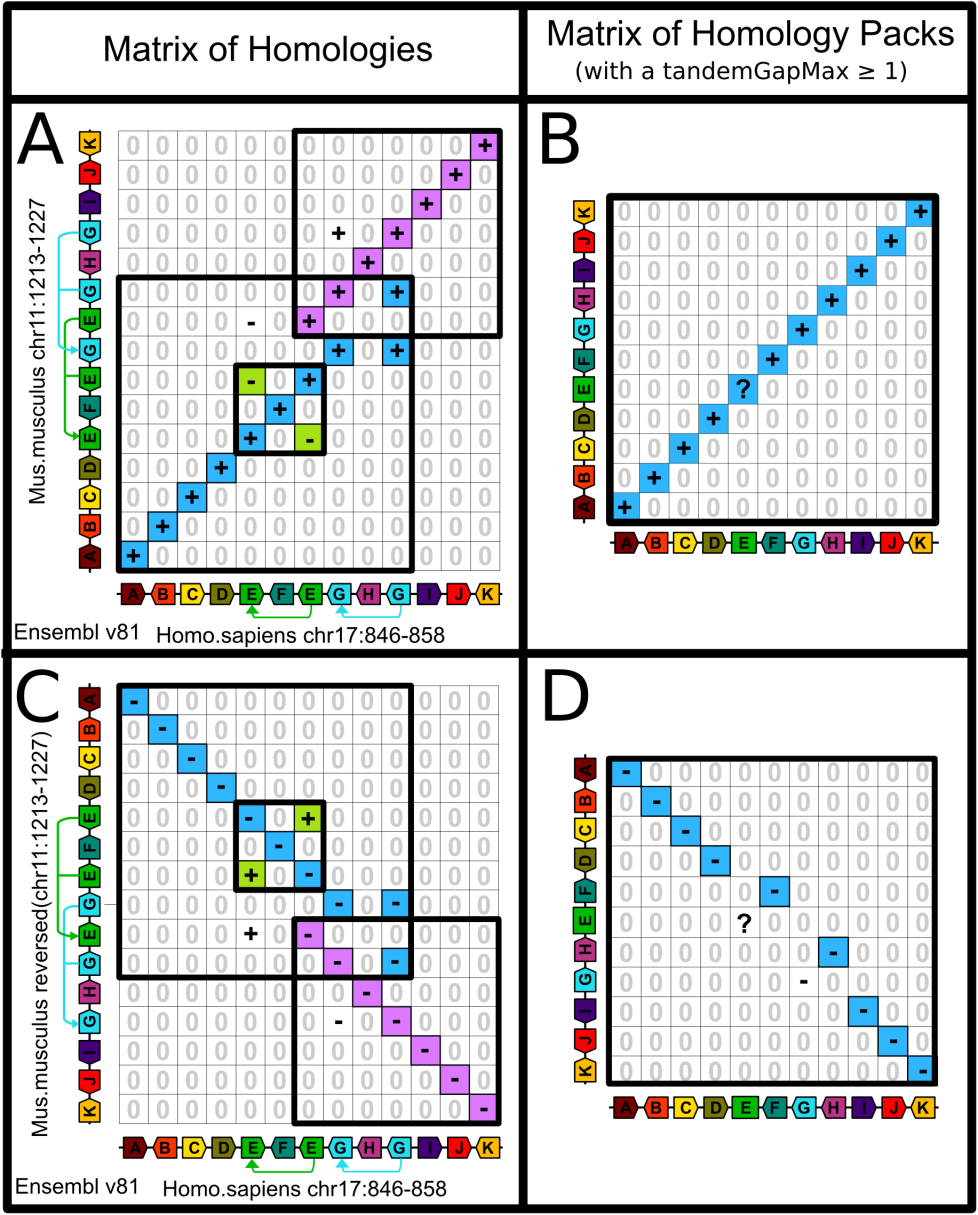
Chromosomes orientations may influence conserved segments identification when tandem duplicates are collapsed. Panel **A** shows the matrix of homologies of the comparison of a segment of human chromosome 17 and a segment of mouse chromosome 11 from Ensembl v81. For a *tandemGapMax* ≥ 1, the human segment contains two clusters of tandem duplicates, one with two genes E and one with two genes G, while the mouse segment also contains two clusters of tandem duplicates for E and G but with three copies of each. The matrix of homology packs [5] after collapsing all clusters is shown in panel **B**. Panel **C** shows the same data as in panel A, but this time the mouse segment is inverted on the y-axis, so that now mouse genes are ranked in the opposite order. With the new orientation of the mouse segment, the gene content of the resulting conserved segment (ABCDEFGHIJK in Panel A and ABCDFHIJK in Panel D) changes but in both cases the two extremities are the same, the 5’ extremity of the gene A and the 5’ extremity of gene K.

Because the representative locations of clusters are arbitrarily assigned to the index of their first tandem duplicate, the result of the rewriting may vary depending on the orientation of the chromosome, that either ranks genes in one order or the reverse (Fig 4).

If clustered paralogs have the same orientation, the representative orientation of the cluster is equal to the consensus orientation (either +1 or -1). For instance, in Fig 3, the representative orientation of the three C genes is +1 and the representative orientation of the three D genes is -1. In contrast, if at least two genes of a cluster of tandem duplicates have different orientations, the representative orientation is considered “unknown” and the orientation is equal to a special value, ∅. We explained in [5] how extended definitions of homology matrices and diagonals encompass “unknown” orientations. For simplicity we will consider here that all clusters of tandem duplicates have a known representative orientation, either +1 or -1.

When comparing two extant genomes, collapsing clusters of tandem duplicates (in addition to removing genes with no homolog in the other genome) brings the matrix of homologies (S7 Fig) closer to the matrix with only conserved ancestral genes (S5 Fig). The remaining difference is at least due to dispersed copies of ancestral genes.

### Post-processing

From now on, we consider that we have identified a set of synteny blocks, in the form of diagonals with gaps ≤ *gapMax*.

#### Identifying micro-rearrangements of at least two genes within gaps of synteny blocks

**Fig 5:**
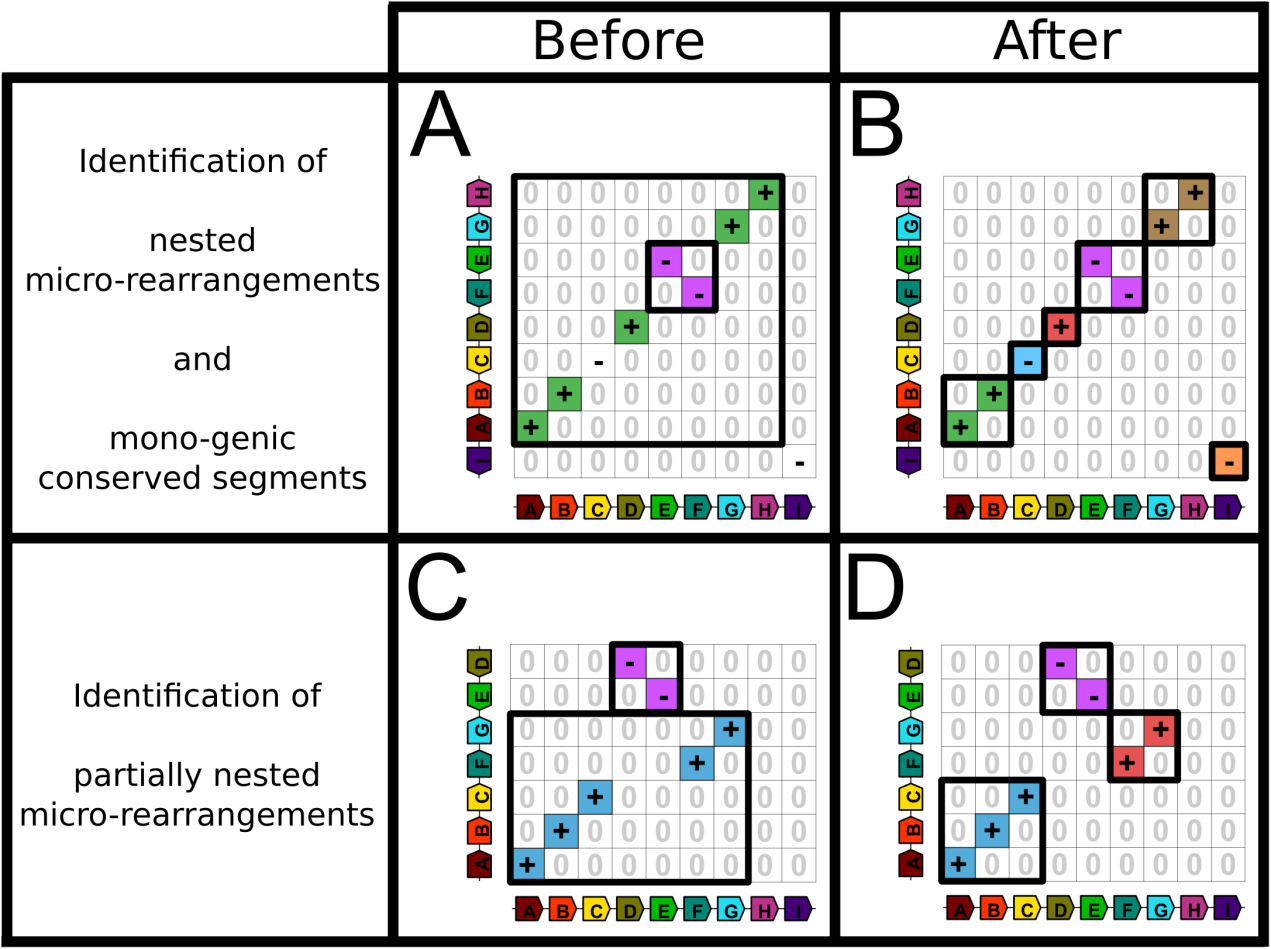
Identifying micro-rearrangements and mono-genic conserved segments. In panel A, the homology matrix corresponds to two synteny blocks, one (corresponding to the purple diagonal) completely nested in the other (green diagonal). In addition, the large diagonal contains a nested single-gene homology (- sign in the middle) and is adjacent to another single-gene homology (the bottom-right—sign). In panel B, after post-processing, the two synteny blocks are now broken down into four conserved segments of which one is mono-genic (corresponding to the red homology) and the two single gene homologies are identified as mono-genic conserved segments (blue and orange homologies). In Panel C, there is a synteny block (purple diagonal) partially nested in another block (blue diagonal). In Panel D, the identification of the corresponding micro-rearrangement leads to three separate diagonals of conserved segments.

To identify micro-rearrangements of at least two ancestral genes, we search for completely or partially nested synteny blocks within gaps of other synteny blocks. We consider that a synteny block A is *completely nested* in a synteny block B if the genes of A are nested in B in both extant genomes (Fig 5A) and *partially nested* if the genes of A are nested in B in only one extant genome (Fig 5C). The host block is thus split at the insertion location of the nested block (Fig 5C and 5D). A consequence of identifying these micro-rearrangements is to increase the overall number of blocks, to decrease the lengths of blocks and also to identify new rearrangement breakpoints. In the next section we will see how to identify the specific case of micro-rearrangements of one unique ancestral gene.

#### Identifying mono-genic conserved segments

To identify mono-genic conserved segments we search for single-gene homologies that are completely nested within the gaps of diagonals with a opposite sign compared to the surrounding signs of the host diagonal (Fig 5A and 5B). Additional mono-genic conserved segments are identified in the neighbourhood around edges of bounding boxes of diagonals and around edges of the homology matrix. For instance, a single-gene homology immediately adjacent to an extremity of a diagonal with opposite signs is identified as a mono-genic conserved segment (orange homology in Fig 5A and 5B).

#### Solving overlaps

**Fig 6:**
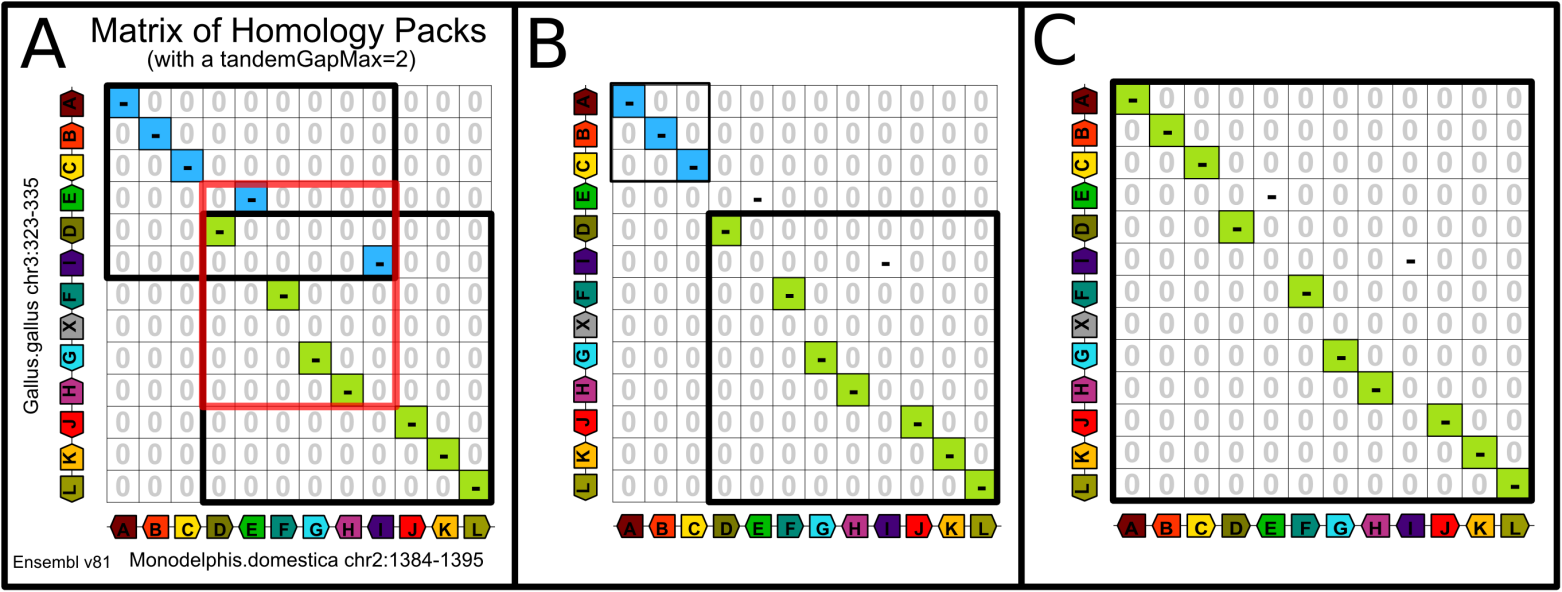
Resolution of overlaps and resolution of what seems to be an incorrect rupture of synteny. The homology matrix in Panel **A** corresponds to the comparison of a segment of the opossum chromosome and a segment of the chicken chromosome with a *tandemGapMax* = 2 in Ensembl version 81. The scenario that leads to the compared extant genomes is debatable. Except for the insertion of the gene X (grey gene on the y-axis) that is probably due to a dispersed duplication, the scenario may involve: inversions, transpositions of genes over a small distance or tandem duplications (with the insertion of copies a few genes away from copied ancestral genes) followed by deletions of the copied ancestral genes. Since tandem duplications and gene deletions seem to outnumber chromosomal rearrangements, we made the choice of considering that the true scenario involves only two tandem duplications followed by deletions of the copied ancestral genes thus both limiting uncertain breakpoints and uncertain extremities of conserved segments. The homology matrix in Panel **B** represents the result of the truncation used to solve small overlaps of diagonals. Panel **C** shows the result of the merge of the extremity of the truncated diagonals that solves the initial incorrect rupture of synteny, delimited by the red rectangle in Panel A.

We now describe a two-stage refinement to solve the problem of overlaps between putative synteny blocks, i.e. overlaps of their diagonals and more precisely the overlap of the orthogonal projections of the bounding boxes of the diagonals. First, for overlaps between two diagonals that are smaller than a user-defined parameter *truncationMax*, the overlapping region containing the fewest homologies is truncated (Fig 6A and 6B). Second, for diagonals that still overlap after the truncation, a subset of non-overlapping diagonals is selected while minimising the number of dismissed homologies in discarded diagonals. To do this we start by creating a conflict graph [21] where vertices are diagonals that have overlaps and where edges are overlaps (conflicts) between pairs of diagonals. Afterwards, the diagonal with the highest number of homologies is selected and the corresponding vertex is removed from the graph. Due to overlaps with the selected diagonal, all the vertices previously connected to the removed vertex are also removed and corresponding diagonals are discarded. Edges are updated and those without two vertices at their extremities are removed. Newly isolated vertices (diagonals with no more overlap) are automatically selected and corresponding vertices are removed from the graph. Then another diagonal with the highest number of homologies is selected. As previously, overlapping diagonals are then discarded and the graph is edited. This recursive procedure is performed until all overlapping diagonals have been selected or discarded, i.e. until the conflict graph is empty. The recursive procedure to solve a conflict graph has been previously explained [21]. The “weighted rectangles” of [21] correspond in our case to bounding boxes of diagonals weighted with the number of homologies in each diagonal.

#### Repairing incorrect ruptures of collinearity by truncating one extremity followed by fusion of the truncated extremity

A local ambiguity on the conservation of the ancestral gene order often yields two incomplete putative synteny blocks, one on each side of the ambiguous region, see Fig 6A. After the truncation explained above, the last refinement step simply merges the extremities of truncated diagonals of synteny blocks, and false conserved segment extremities are then eliminated (Fig 6B and 6C).

The four post-processing steps are integrated into a workflow (S11 Fig) which improved the quality of our results on real data compared to more simple workflows.

### Recall and precision analysis of the detection of conserved segments

The pre-processing step that collapses clusters of tandem duplicates and the four refinement steps have been implemented in a new version of PhylDiag [5] benchmarked by simulations, showing substantial improvements. Using a realistic simulation of the evolution of gene order [9](S1 Text) and the corresponding simulated conserved segments, we computed the recall and precision of this new version of PhylDiag in recovering the true conserved segments. Although they are not *strictly* conserved segments, the synteny blocks of i-ADHore 3.0 and Cyntenator (more precisely the base_clusters of i-ADHoRe 3.0 and the RSBs of Cyntenator) are also included in the benchmark as if they were conserved segments, since, to our knowledge, they are the most relevant entities to approach our definition of conserved segments. For this reason we will compare the synteny blocks of i-ADHoRe 3.0 and Cyntenator in the same conditions as the conserved segments of PhylDiag. Furthermore, identifying accurate synteny blocks (resp. conserved segments) from a comparison of two genomes is a prerequisite for the identification of accurate synteny blocks (resp. conserved segments) from a multi-genomes comparison, thus we only compare pairs of genomes in the benchmark, although, contrary to PhylDiag, i-ADHoRe 3.0 and Cyntenator allow multi-genome comparisons. We used base_clusters of i-ADHoRe 3.0 instead of multiplicons because base_clusters seemed closer to our definition of conserved segments. Similarly, we wanted to use Conserved Syntenies over Multiple species (CSMs) from Cyntenator, [7], that are probably closer to our definition of conserved segments. However, the output only returned RSBs, a RSB being the largest genomic region of all overlapping CSMs.

**Fig 7:**
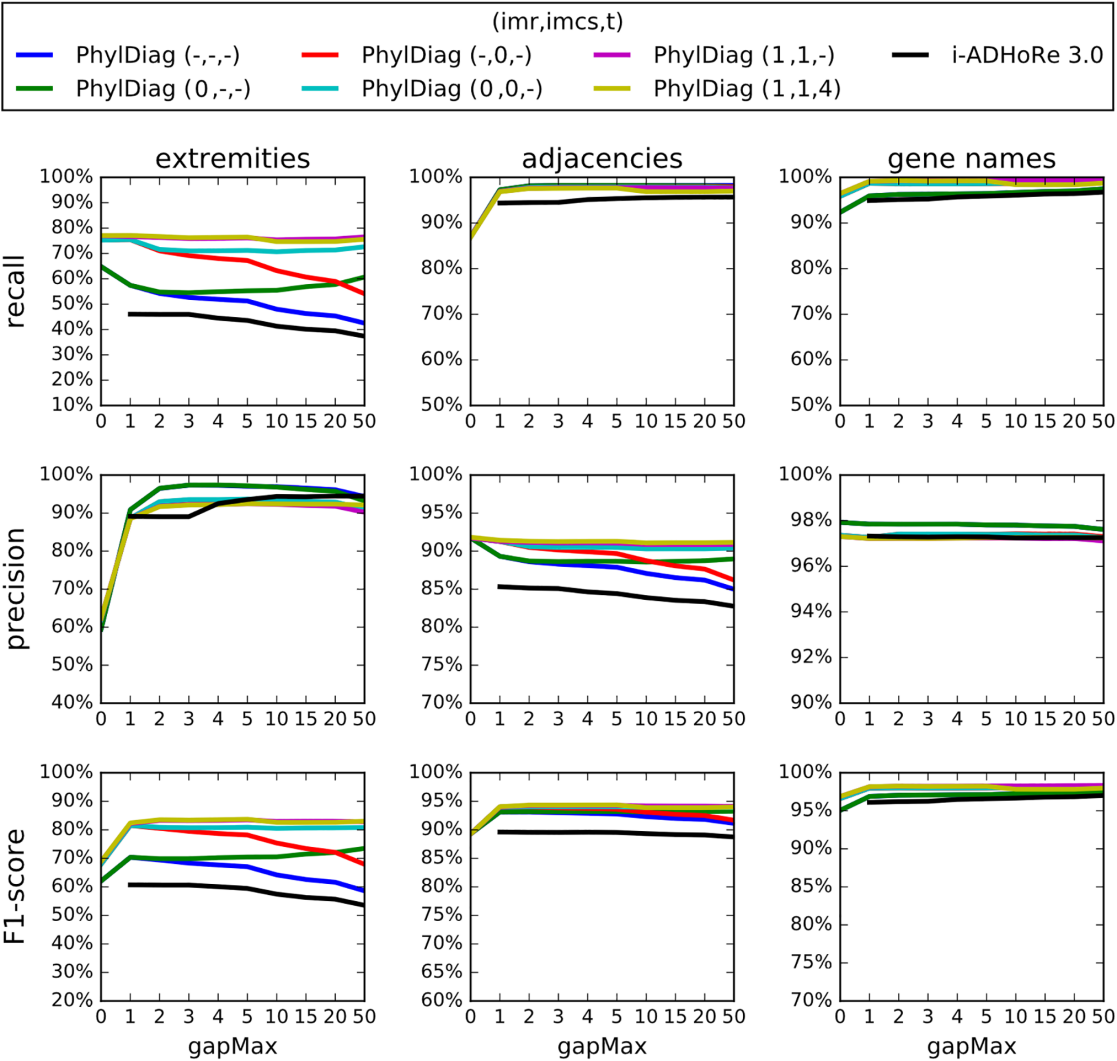
Recall and precision analysis of PhylDiag and i-ADHoRe 3.0 based on simulated conserved segments of two distant species, mouse and chicken that diverged 325 Million years ago. The analysis is performed based on a realistic simulation [9](S1 Text) of the evolution of gene order that replicates features of extant genomes of Ensembl 81. The first column (left) corresponds to the detection of extremities of conserved segments. The second column (middle) corresponds to the detection of adjacencies of genes in conserved segments. And the third column (right) corresponds to the detection of gene names in conserved segments. For each item and each parameterisation of algorithms, recall (top), precision (middle) and F1-score (bottom) are shown as a function of *gapMax*. The refinement methods described in this manuscript are *imr* (Identify Micro-Rearrangements), *imcs* (Identify Mono-genic Conserved Segments) and *t* (Truncation). A “-”sign means that the option is inactive and an integer, even 0, means that the option is active. For the option *imr*, the integer value specifies the maximum gap allowed between: the extremity of an identifiable micro-segment and the nearest homology of the diagonal in which it is included (S12 Fig). For the option *imcs*, the integer value sets the width of the neighbourhood around edges of bounding boxes of diagonals of synteny blocks where mono-genic conserved segments are identified (S13 Fig). For the option *t*, the integer value specifies the *truncationMax* parameter value. Truncating and solving remaining overlaps with *truncationMax* = 4 does not decrease substantially recall and precision while ensuring that conserved segments are not overlapping. The black curves represent the results of i-ADHoRe 3.0 for varying values of the parameters *gap_size* and *cluster_gap*, both equal to *gapMax* along the axis. *gap_size* and *cluster_gap* parameters of i-ADHoRe 3.0 does not allow 0 values thus graphs of i-ADHoRe 3.0 begin at gapMax = 1.

**Fig 8:**
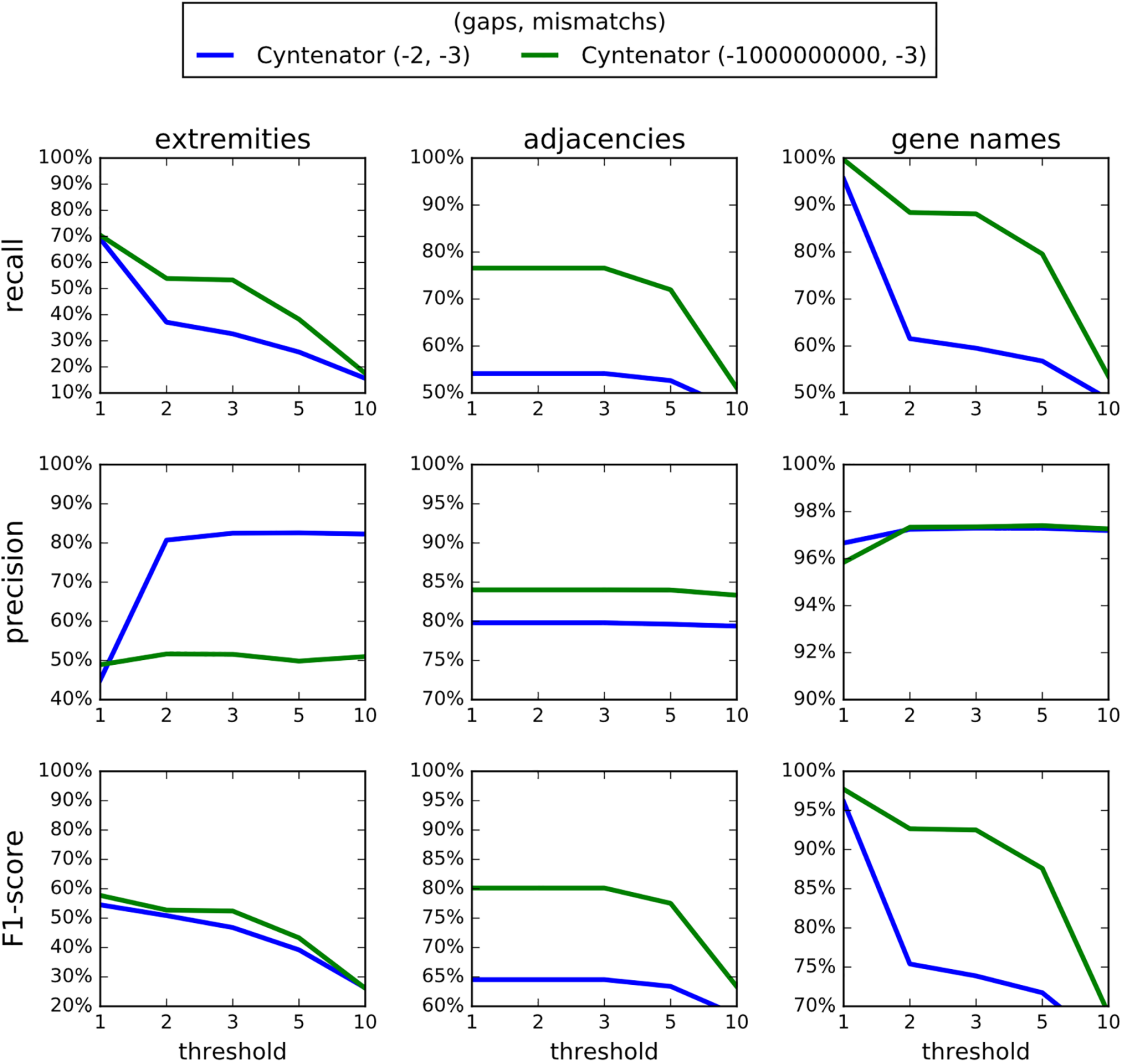
Recall and precision analysis of Cyntenator based on the same simulation as in Fig 7. The parameter varying is *threshold*, the cut-off value of Cyntenator that discards all alignments of genes with lower scores.

More precisely, we set some parameters of i-ADHoRe 3.0 to their default value: *anchor_points* = 3, *prob_cutoff* = 0.001 and *tandem_gap* = 5 (equal to the value of the parameter *tandemGapMax* we used for PhylDiag). In Fig 7, the remaining parameters *gap_size* and *cluster_gap* are equal and vary as the values of the corresponding parameter *gapMax* in PhylDiag. Different values of the probability threshold (*proba_cutoff*) have been tested and we kept the value that maximised the results of i-ADHoRe 3.0, corresponding to the default value. We used the default values for the parameters of Cyntenator: *mismatch* = -3, *coverage* = 2 and *filter* = 100 except for the parameters, *threshold* and *gap* that varies (Fig 8). We also tried several *mismatch* values but they did not change substantially the results and corresponding graphs almost completely overlapped graphs with the same *gap* value in Fig 8 so we omitted them. Pre-processing genomes and collapsing clusters of tandem duplicates (still with *tandemGapMax* = 5), did not change significantly the results of Cyntenator. Figs 7 and 8 can be reproduced following the protocol dx.doi.org/10.17504/protocols.io.idnca5e.

The quality of the detection of conserved segments is estimated for each algorithm using three types of items: extremities of conserved segments, adjacencies of genes in conserved segments and gene names in conserved segments. For one simulation, the set of detected items (*D*) derived from conserved segments inferred from extant simulated genomes is compared to the set of true items (*T*) derived from conserved segments recorded during the simulation. The set of true positive items (*Tp*) contains the intersection of both sets, *Tp* = *D* ∩ *T*. The set of false positive items (*Fp*) contains the set of detected items that are not true, *Fp* = *D* \ *T*. The set of false negative items (*Fn*) is the set of true items that are not detected *Fn* = *T* \ *D*. The recall (*r*), also called sensitivity, and the precision (*p*) are defined by the equations

$$r=\frac{\left|Tp\right|}{\left|T\right|}=\frac{\left|Tp\right|}{\left|Tp|+|Fn\right|}\;\mathrm{and}\;p=\frac{\left|Tp\right|}{\left|D\right|}=\frac{\left|Tp\right|}{\left|Tp|+|Fp\right|},$$

with |*x*| the number of items in the set *x*. We also calculated the F1-score (*F*1), also called F-score or F-measure. It is a common tradeoff between recall and precision, giving equal importance to both and it may thus be considered as a general score for quantifying the quality of detection. The F1-score is the harmonic average of recall and precision,

$$F1=\frac{2\cdot r\cdot p}{r+p}.$$

**Fig 9:**
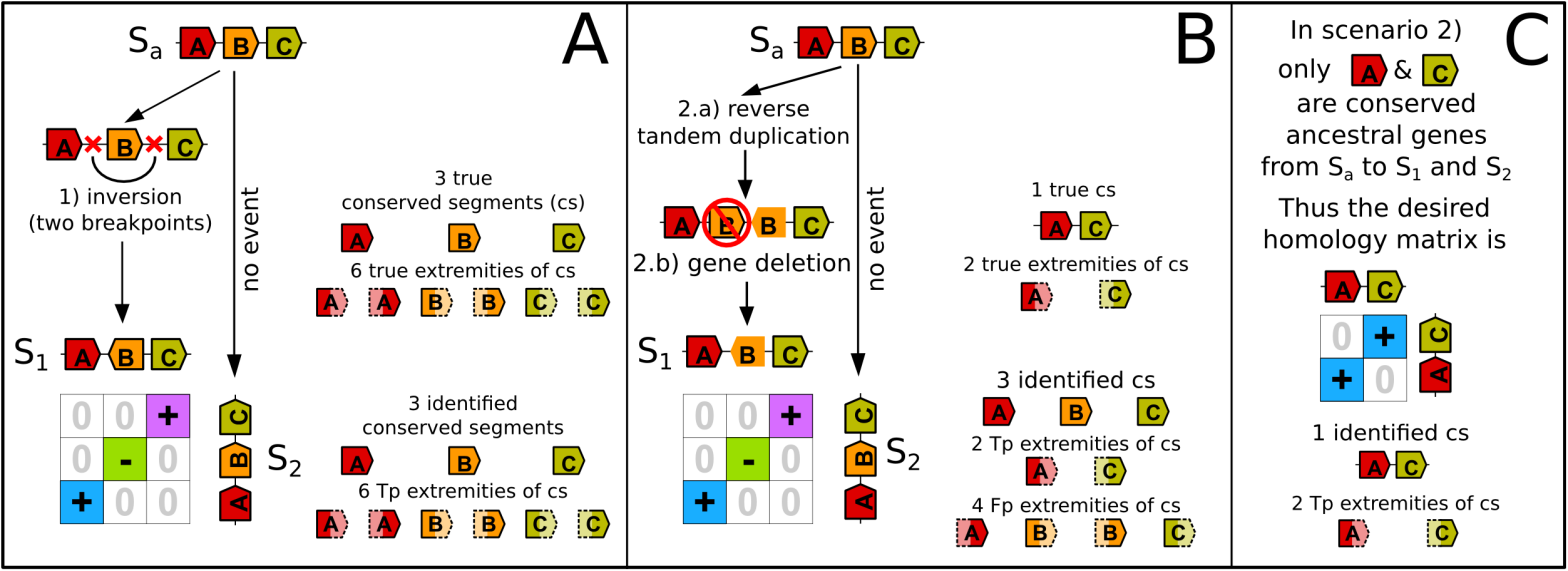
Two scenarios, one with breakpoints, the other without breakpoint that cannot be distinguished with our data. In Panel **A**, an initial chromosome of an ancestral species S_**a**_ evolves up to two extant species, S_**1**_ and S_**2**_. No events take place from S_**a**_ to S_**2**_ but gene B is inverted between S_**a**_ and S_**1**_, creating two breakpoints and resulting in three conserved segments which are easily identified in the homology matrix at the bottom. In Panel **B**, gene B is tandemly duplicated with a reverse orientation from S_**a**_ to S_**1**_, and the ancestral copy is deleted. From the comparison of extant genomes of S_**1**_ and S_**2,**_ since the non-ancestral gene B (with no black outer line) is incorrectly considered as an ancestral gene, the homology matrix appears identical to the mono-genic inversion scenario in panel A. Therefore 3 conserved segments are returned and 6 extremities of conserved segments are detected whereas only one conserved segment should be returned, with two extremities, as explained in Panel **C**. In Panel B, the sets of extremities of conserved segments used for the calculation of the recall and the precision are T = {sA,eC}, D = {sA,eA,sB,eB,sC,eC}, Tp = {sA,eC}, Fp = {eA,sB,eB,sC} and Fn = ∅; with sX the 5’ extremity (start) of the gene X and eX the 3’ extremity (end). Thus the recall is 100% and the precision is 33%. Similarly, if gene orientations are not considered, T = {A,C}, D = {A,B,C}, Tp = {A,C}, Fp = {B} and Fn = ∅, thus the recall is 100%, the precision is 66%. Concerning gene adjacencies with gene orientations, T = {eA-sC}, D = ∅, Tp = ∅, Fp = ∅ and Fn = {eA-sC}; with X-Y the adjacency of the gene extremity X and gene extremity Y. Thus recall and precision are both null here. Finally, if we are interested in gene names in conserved segments, T = {A,C}, D = {A,B,C}, Tp = {A,C}, Fp = {B} and Fn = ∅ thus the recall is 100% and the precision is 66%.

**Fig 10:**
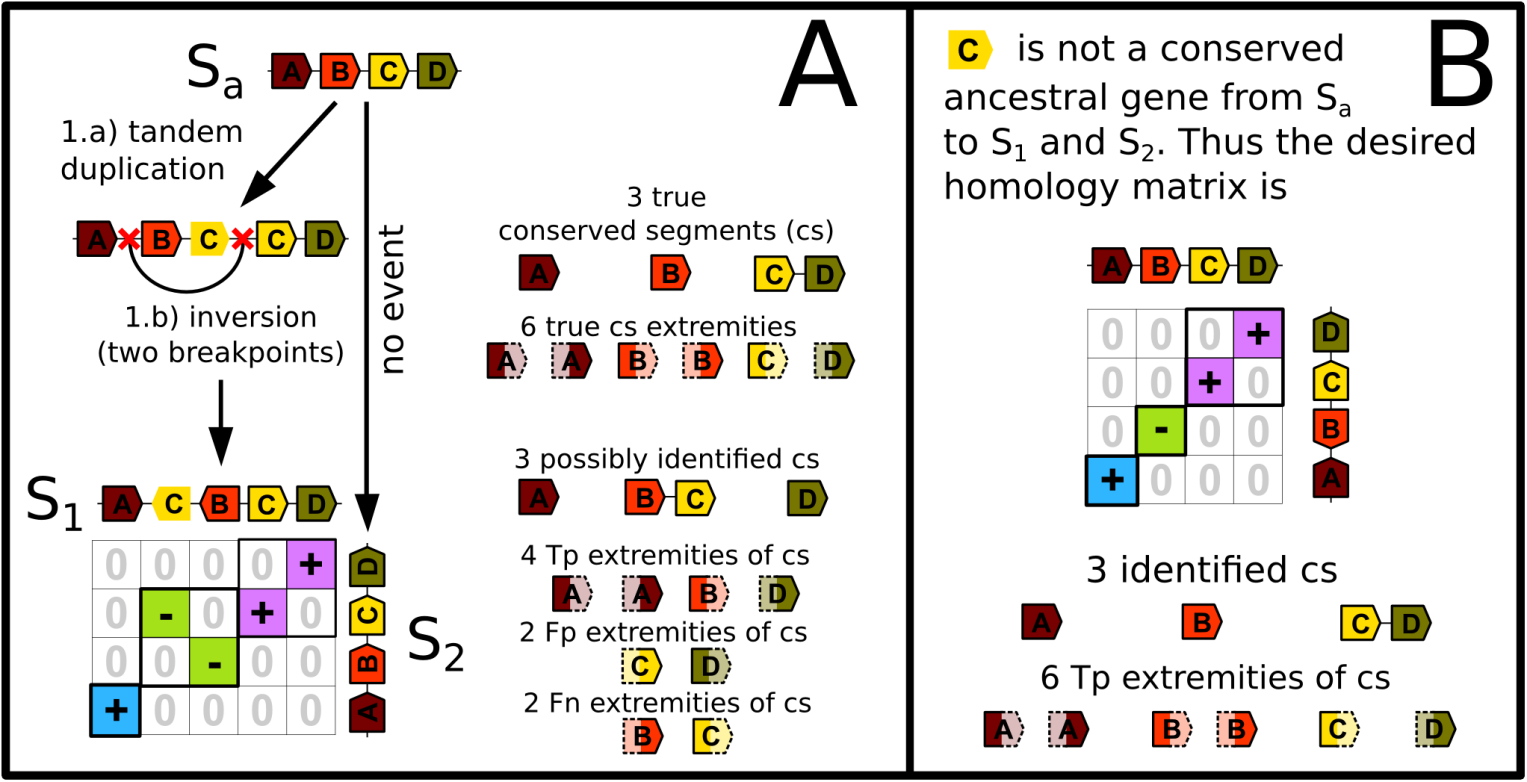
Breakpoints between tandem duplicates yield unsolvable false positive and false negative extremities of conserved segments. In Panel **A**, an initial chromosome of an ancestral species S_**a**_ evolves until two extant species, S_**1**_ and S_**2**_. Along the lineage from S_**a**_ to S_**2**_ the chromosome is perfectly conserved, and along the lineage from S_**a**_ to S_**1**_ gene C is duplicated in tandem. The non-ancestral copy of gene C has no black outer line. Then an inversion occurs with the right breakpoint falling between the two tandem duplicates. From extant genomes of S_**1**_ and S_**2**_ it is impossible to know which paralog of gene C in S_**1**_ is non-ancestral, thus both copies are considered as a probable ancestral gene C. Although the analysis of the homology matrix yields 3 conserved segments, if the non-ancestral gene C is falsely considered as the ancestral gene, there are two false positive extremities and two false negative extremities. Panel **B** describes the desired homology matrix obtained when the ancestral gene C is correctly identified. In Panel A, the sets of extremities of conserved segments used for the calculation of the recall and the precision are T = {sA,eA,sB,eB,sC,eD}, D = {sA,eA,sB,eC,sD,eD}, Tp = {sA,eA,sB,eD}, Fp = {eC,sD} and Fn = {eB,sC} with sX the 5’ extremity (start) of the gene X and eX the 3’ extremity (end). Thus the recall and the precision are both equal to **66%**. If we focus on the detection of gene adjacencies without considering gene orientations, T = {C-D}, I = {B-C}, Tp = ∅, Fp = {B-C} and Fn = {C-D} with X-Y the adjacency of genes X and Y. The associated recall and precision are thus null here.

Examples of true positives sets of detected items, false positives sets, false negatives sets and sets of true items are given at the end of the captions of Figs 9 and 10; followed by the corresponding recalls and precisions.

To make the comparisons possible with i-ADHoRe 3.0 and Cyntenator, we did not account for gene orientations in conserved segments in our calculations, i.e. extremities of conserved segments are the gene names only and adjacencies are pairs of gene names.

First, we focus on the detection of extremities of conserved segments with the following options activated in PhylDiag: detection of mono-genic conserved segments, detection of micro-rearrangements and resolution of small gene order disruption (yellow curve). Results show a 20% increase in recall at *gapMax* = 1 from 57% up to 77% without any substantial decrease in precision (Fig 7). As explained later in Figs 9 and 10, precision is high but never reaches 100%, which is most likely due to the numerous gene duplications and deletions that occurred in the mouse and chicken lineages that raise the number of false positives. For all *gapMax* values the recall and precision of i-ADHoRe 3.0 are lower or close to the recall and precision of PhylDiag, even without any post-treatment (blue curve) (Fig 7). The results of Cyntenator are displayed in Fig 8, since it has no parameter equivalent to the *gapMax* parameter the varying parameter is *threshold*, i.e. the main parameter of Cyntenator representing a cut-off value that discards all segments with lower scores. For all values of the parameter *threshold* tested, either the precision is less than 60% or the recall is less than 60%: the increase in precision gained by increasing *threshold* is compromised by a drastic loss of recall.

Second, if we consider gene adjacencies rather than extremities, the recall and precision of PhylDiag is still above the recall and precision of i-ADHoRe 3.0 whatever the *gapMax*. The analog recall and precision of Cyntenator both stay under 87%, below the recall and precision of PhylDiag reached with most of the *gapMax* values.

Finally, if we study the detection of gene names, the precision of PhylDiag is higher or close to the precision of i-ADHoRe 3.0 as soon as at least one of the options is activated, while the recall of PhylDiag stays substantially 3–4% above. The recall of Cyntenator is very high (99.66%) with a *threshold* equal to 1 and a *gap* equal to -1000000000 although the corresponding precision (95.84%) is below the precision of PhylDiag (above 97% whatever the activated options).

F1-scores show that PhylDiag is always ahead whatever the type of items considered and whatever the options activated, except in a marginal case: when comparing ancestral families, Cyntenator, with a deterrent gaps cost (*gap* = -1000000000) and a *threshold* equal to 1 reaches a higher F1-score than PhylDiag.

Poor F1-scores of Cyntenator when considering extremities of conserved segments or adjacencies of genes might be explained by considering RSBs’ components: CSMs, that are probably closer to our definition of conserved segments. The integration of overlapping CSMs within long RBSs probably leads to numerous false negative extremities and false positive adjacencies. Extremities of a CSM corresponding to a true conserved segment will be overlooked, if it is embedded within a RSB. In addition, the junction of neighbouring CSMs embedded in the same RSB will be detected as an adjacency of the RSB, leading to false positive adjacencies as soon as both embedded CSMs are genuine conserved segments.

Fig 7 also shows that, with PhylDiag, a *gapMax* equal to 0 returns low F1-scores because of artificial gaps due to dispersed duplications creating scattered artificial gaps equal to 1 gene. On the contrary a *gapMax* of 1 is enough to reach optimal F1-scores since only dispersed duplications and no other source of artificial gaps are simulated. Despite this, experience shows that a higher *gapMax* is required for returning accurate conserved segments when comparing real genomes, since assembly and annotation errors generate additional artificial gaps in conserved segments. Fortunately, when studying extremities and gene adjacencies of conserved segment, activating the detection of micro-rearrangements strikingly stabilises the recall of the detection of extremities and the precision of adjacencies. Thus even high *gapMax* values can be used with lasting high recall and lasting high precision; contrary to corresponding recalls and precisions of i-ADHoRe 3.0 that both decrease when the *gapMax* increases, due to the presence of micro-rearrangements within base_clusters.

### Justification of remaining false negatives and false positives

Using simulated conserved segments makes it possible to investigate the source of remaining mistakes during the detection of extremities of conserved segments: false positive and false negative extremities of conserved segments, which prevent the recall and precision of PhylDiag to reach 100% (Fig 7). Based on our simulations, it appears that mistakes come mainly from scenarios involving ancestral genes, first duplicated in tandem and then deleted, and also from breakpoints within clusters of tandem duplicates. We estimate these two scenarios to be very common in our simulation (S15 Fig). Two examples of these types of scenarios are depicted in Figs 9 and 10. The first corresponds to extremities of mono-genic segments, which appear as mono-genic inversions but which in fact result from a reverse tandem duplication [18] followed by a mono-genic deletion (Fig 9). Both situations lead to indistinguishable gene arrangements in extant genomes. Our process of identifying mono-genic conserved segments always favours the detection of mono-genic inversions (Fig 9A) instead of the less parsimonious but rearrangement-free duplication-deletion scenario (Fig 9B). Hence our observation that recall increases when the mono-genic inversion occurred and that precision decreases when the rearrangement-free scenario occurred.

A second source of false positive and false negative extremities of conserved segments are breakpoints that fall between tandemly duplicated genes (Fig 10).

Knowing which tandem duplicate is the ancestral gene would solve wrong identifications explained in Figs 9 and 10 but since the two tandem duplicates play a symmetrical role after the duplication, it is impossible to distinguish the ancestral gene. Thus evolutionary scenarios similar to those of Figs 9 and 10 yield indiscernible extremities of conserved segments that prevent recall and precision to be 100%. In both scenarios depicted here, the ancestral genome is altered within one unique lineage. Similar scenarios where the ancestral genome is altered in both lineages, leading to unavoidable wrong identifications probably occur, but seem less frequent from our non-exhaustive analysis. In addition, since our simulator may not realistically simulate breakpoints reuse [22], we were not able to estimate how “done and undone rearrangements” (for example two successive inversions that reverse the same segment twice) are substantial sources of false negatives.

## Conclusion

In the field of comparative genomics performed at the scale of whole genomes, small rearrangements are generally dismissed from consideration because they are difficult or impossible to distinguish from assembly and annotation errors [2]. Yet the corresponding breakpoints of true small inversions may completely reshape a set of conserved segments that was detected without considering them. As a consequence, what may be thought to be a long conserved segment may prove to be several conserved segments of modest sizes when micro-inversions are considered. With improvements in the accuracy of genome assemblies and annotations, we believe that it might be time to start studying small rearrangements simultaneously to macro-rearrangements. In this context our method is the first to identify conserved segments up to mono-genic segments. It finds conserved segments with a level of accuracy that takes the full advantage of knowing the transcription orientations of genes (Fig 5). Furthermore it solves complex cases of synteny involving remnants of tandem duplications (Figs 3 and 4) as well as incorrect ruptures of syntenies (Fig 6). Yet we show that the quality of conserved segment detection cannot reach 100% since some scenarios of evolution without breakpoint yield similar extant genomes to scenarios of evolution with breakpoints (Figs 9 and 10).

We also show that modular pre-processing of genomes and post-processing refinements of synteny blocks, with few parameters, mainly *tandemGapMax* and *truncationMax*, represent an intuitive yet rigorous alternative to all-in-one greedy graph-based heuristics [19,20,23] that may be more difficult to configure [24]. In addition, it has been demonstrated [24] that structures of such graphs “do not show crucial pieces of alignment information”. For instance, except for Enredo[19], they do not display mono-genic inversions.

Here, we considered chromosomes as linear arrays of ordered and oriented genes, but considering nucleotide sequences could help resolve some remaining issues. For example, it may help distinguish the ancestral gene among clusters of tandem duplicates. This would provide a more accurate localisation of the ancestral gene copy within a cluster of tandem duplicates and would help identify the correct scenario in Figs 9 and 10. Nucleotide sequences could also be used to extend conserved segment extremities in order to further investigate precisely delimited breakpoint regions as in [16]. Also, when solving the conflict graph, the method currently implemented in PhylDiag may return a suboptimal set of non-overlapping diagonals and another set of non-overlapping diagonals may exist with more homologies [21].

In line with a previous comparison [8], ours revealed substantial differences between algorithms aiming at identifying “synteny blocks”, even while comparing two genomes instead of many genomes. We confirm that the community would benefit from a clear definition of a “synteny block”, and we provide and exploit here an evolutionarily consistent definition that follows from the intuitive definition of Pevzner and Tesler [2]. It would be interesting to investigate if the pre- and post-processes ideas presented in this article could improve multi-genome comparisons. Improved accuracy of multi-genome conserved segments would be greatly beneficial for the inference of rearrangements scenarios from one ancestral genome to more than two genomes.

Finally, it would be of great interest to simulate errors (assembly errors, annotation errors or even errors in gene trees) in addition to the main known events that alter genomes during evolution (S14 Fig). This could help us understand how these errors affect algorithms–given that some algorithms might be more robust to errors than others. However, relevant statistics on errors in genomic data (e.g. vertebrate genomes of the Ensembl database) are difficult to obtain. We hope that an increased consideration of micro-rearrangements will help detect such remaining errors.

## Supporting information

S1 Fig Evolution from of an ancestral genome made of 2 chromosomes (top) along two lineages, to two extant species S_1_ and S_2_. The arrow in the center, pointing towards the bottom, is the time arrow. The genome before and after each event is drawn to show how each event altered it. Colours and capital characters correspond to gene families. Genes outlined in black are ancestral genes except genes filled in white that are genes from families originating after the speciation (genes specific to one lineage). Genes not outlined in black are non-ancestral genes inserted due to duplication. Small characters after dots help here to differentiate copies and copied genes: A.a is not the same gene as gene A even if they are both in the family of gene A. Furthermore if gene A was once more duplicated there would be an instantiated copy named A.b newly inserted in the genome and if the gene A.a was duplicated there would be an instantiated copy named A.a.a. The dates of the events, that either include chromosomal rearrangements with breakpoints or ancestral gene deletions, are specified along the time arrow because they alter conserved segments. Chromosomal fusions, gene duplications and *de novo* gene births do not alter conserved segments. Corresponding evolutions of conserved segments along different lineages are drawn afterwards in S2 and S3 Figs.
(PDF)

S2 Fig Evolution of the conserved segments from the ancestral genome *to S*_*1*_ corresponding to the evolution depicted in S1 Fig. Between t = 0 and t = t_1_, before any alteration of the ancestral gene order or ancestral gene content, the conserved segments are exact copies of the ancestral chromosomes in the ancestral genome. The two breakpoints of the translocation 1 (in the first lineage) start breaking the conserved segments at the two corresponding spaces between ancestral genes. Afterwards, the gene deletion 1 removes the ancestral gene G from the conserved segments. Next, breakpoints associated with extremities of rearranged segments keep fragmenting conserved segments until the 7 final conserved segments from the ancestral genome to S_1_.
(PDF)

S3 Fig Evolution of the conserved segments from the ancestral genome *to S*_*1*_ *and S*_*2*_ corresponding to the evolution depicted in S1 Fig. Here again, before the translocation 1 the conserved segments are exact copies of the ancestral chromosomes, and the translocation 1 (in the first lineage) starts breaking the conserved segments at two breakpoints. The next event altering conserved segments along both lineages is, in this case, an event in the second lineage: fission 2. Considering events in both lineages returns more and smaller conserved segments than in S2 Fig: here, when the evolution is finished, 10 segments of the ancestral genome have been conserved. In addition, more deletions of ancestral genes in conserved segments are expected when studying the conservation of ancestral segments in multiple lineages instead of in a unique lineage. For instance, the deletion of the ancestral gene G causes the loss of the ancestral gene G, in conserved segments from the ancestor to S_1_ and S_2_, even if the deletion only occurred in one lineage; same for the deletion of the ancestral gene F.
(PDF)

S4 Fig Matrix of homologies of the comparison of the genome of S_1_ (on the x-axis) and the genome of S_2_ (on the y-axis). Each chromosome on the x-axis is ordered from left to right: it starts at left and ends at right. On the y-axis chromosomes are ordered from bottom to top. A gene has a positive orientation if its 3’-5’ orientation (its arrow here) points to the end of its host chromosome. In the contrary, it has a negative orientation if it points to the beginning of the chromosome. For instance, gene P of S_1_, gene P of S_2_ and gene E of S_2_ have positive orientations (they point either to the right or to the top) whereas gene E of S_1_ has a negative orientation, it points to the left. The matrix is an array of *signs* equal to +, − or 0. Non-0 signs correspond to *homology signs*. For instance, the homology sign corresponding to gene P in S_1_ and gene P in S_2_ is “+” because both genes have a positive orientation. In contrast, Gene E in S_1_ has a negative orientation and Gene E in S_2_ has a positive orientation thus the corresponding homology has a “-” sign. In the matrix, diagonals of conserved segments from the ancestor to S_1_ and S_2_ are outlined with black rectangles, and homologies within the same diagonal have the same colour. Both compared genomes have 2 chromosomes and the matrix is thus composed of 4 sub-matrices of homologies of the comparison of pairs of chromosomes. Remark: When the evolution from the ancestor to compared extant genomes is unknown, genes of extant genomes cannot be labelled with ancestral gene names and names of copies. Homology relationships between genes are usually estimated from comparisons of nucleotide sequences and, in practice, genes are labelled with family names. Consequently, ancestral gene localisations are often unknown and a localisation of an ancestral gene is often mixed among the locations of its copies. For instance, if we did not known the evolution depicted in S1 Fig, genes B and B.a would both be labelled with the name of their common family, B. Similarly, in Fig 4A of the main manuscript, two genes are labelled E because both genes are in the same family and, before any analysis of the collinearity, may both be the ancestral gene at the root of their family.
(PDF)

S5 Fig Matrix of homologies of two extant genomes that have been perfectly filtered, such that only ancestral genes conserved in both lineages are kept. In this matrix, conserved segments from the ancestor to the two compared extant genomes can be identified as perfect diagonals with no gaps in the matrix of homologies. Remark: When compared genomes are not filtered (S4 Fig), diagonals of conserved segments are blurred with artificial gaps. Dispersed duplications (A.a and A.b), *de novo* gene births (X and Y) and ancestral gene deletions (G and K) create artificial gaps within diagonals (or around diagonals) of conserved segments that would not exist if genomes were perfectly filtered (S5 Fig). Furthermore if compared genomes are not filtered, diagonals of conserved segments are also blurred with artificial packs of homologies, vertical or horizontal (S5 Fig) or even rectangular (see later in S8 Fig) caused by clusters of tandem duplicates. S6 and S7 Figs will give examples of matrices of homologies that are obtained with two pre-processings of compared genomes that gradually approach the perfect filtering.
(PDF)

S6 Fig Matrix of homologies of both extant genomes filtered in such a way that genes without homologs in the other genome are removed. Keeping only genes with homologs in the compared genome removes artificial gaps due to *de novo* gene births and gene deletions but can neither solve artificial gaps due to dispersed duplications (A.a and A.b) nor packs of homologies caused by clusters of tandem duplicates (T and T.a or B and B.a).
(PDF)

S7 Fig Matrix of homologies of both extant genomes filtered in such a way that genes with no homolog in the other genome are removed and with collapsed clusters of tandem duplicates. Collapsing clusters of tandem duplicates in addition to removing genes with no homolog in the other genome bring the matrix of homologies closer to the ideal matrix of homologies with perfectly filtered genomes (S5 Fig). Yet diagonals still contain artificial gaps caused by dispersed duplications, even with this intense filtering. Hence, in the absence of a better way to pre-process compared genomes, algorithms aiming at identifying synteny blocks or conserved segments, have to deal with artificial gaps; at least unitarian gaps scattered in genomes because of dispersed duplications. A *gapMax* = 0 won’t be sufficient to overcome these artificial gaps and values of *gapMax* at least equal to 1 are necessary.
(PDF)

S8 Fig Pruning phylogenetic gene trees help distinguishing lineages of tandem duplicated genes. An ancestral chromosome of three genes ABC evolves with 2 different scenarios until 2 extant species S_1_ and S_2_. In the first scenario, **left column**, the gene B is duplicated in tandem, before the speciation, i.e. the duplication happens between the initial genome and the most recent common ancestor of S_1_ and S_2_, MRCA(S_1_, S_2_). The conserved segment from the MRCA to S_1_ and S_2_ is thus made of 4 ancestral genes. Pruning the gene tree of gene B at the level of the MRCA divides the family of gene B into two families: a smaller family of gene B and a new family corresponding to the legacy of gene B.a. The differentiation of families of genes B and B.a makes it possible to identify the true 4 ancestral genes in the segment conserved from the MRCA to S_1_ and S_2_. The second scenario, **right column**, has two duplications after the speciation that give rise to a similar configuration of tandem duplications in extant species. Contrary to the previous scenario, here, both duplications in tandem happen after the MRCA and insert non-ancestral copies of the gene B: B.a and B.b. There are thus only three genes, ABC, in the conserved segment from the MRCA to S_1_ and S_2_. Pruning the gene tree of gene B does not change the family of gene B in this case since at the level of the MRCA only gene B exists. Thus tandem duplicates are visible in the matrix of homologies and collapsing clusters of tandem duplicates leads once more to the true number of 3 ancestral genes in the detected segment conserved from the MRCA to S_1_ and S_2_. If the scenario of the right column happened, and if the phylogenetic algorithm used for inferring gene trees made a mistake, and found the gene tree of the left column, our detection of conserved segments, as diagonals in the matrix of homologies would make an error. It would detect 4 ancestral genes in the segment conserved from the MRCA to S_1_ and S_2_ instead of the 3 true ancestral genes. Such interdependences between gene trees and collinearity have been used to improve gene trees using PhylDiag [25].
(PDF)

S9 Fig A matrix of homologies corresponding to pre-processed chromosomes of mouse and chicken (chromosomes have been truncated). Both chromosomes contain only genes that have homologs in the other genome and clusters of genes duplicated in tandem have been collapsed. Blue arrows point towards examples of artificial gaps within diagonals of conserved segments. These artificial gaps seem to be due to dispersed duplications or errors (assembly errors, annotation errors or errors in gene families). A zoom of the region circled in red is also shown.
(PDF)

S10 Fig Matrix of homologies calculated with i-ADHoRe 3.0 where a segment of pre-processed human chromosome X is compared to a segment of pre-processed mouse chromosome X. Yellow dots represent homologies in a base_cluster and blue dots represent the “confidence intervals” around the base_cluster, see the documentation of i-ADHoRe 3.0 [6]. Here also, after the pre-processing of i-ADHoRe 3.0 (filter + collapse of clusters of tandem duplicates), artificial gaps remain within a diagonal of a conserved segment.
(PDF)

S11 Fig Workflow for the detection of conserved segments. The scripts/ folder, in the LibsDyogen deposit, provides tools to prune.nhx gene trees and extract gene families from pruned gene trees. The new version of PhylDiag (from v2.0.0-alpha) includes the pre-processing and post-processing steps (https://github.com/DyogenIBENS/PhylDiag).
(PDF)

S12 Fig Maximum gap allowed between: the extremity of an identified micro-segment and the nearest homology of the diagonal in which it is included. An isolated homology is distant with a maximum gap of 1 (length of the black double arrow with the Chebyshev Distance Metric) with the nearest homology of its surrounding diagonal. If the maximum gap allowed for the identification of micro-rearrangements is at least 1, the isolated homology is identified as a mono-genic conserved segment. Black genes are probable ancestral genes inserted because of dispersed duplications or they might be due to errors in data (annotation errors or errors in families).
(PDF)

S13 Fig Maximum width of the neighbourhood around edges of bounding boxes of synteny blocks where mono-genic conserved segments are identified. An isolated homology is distant with a maximum gap of 1 (length of the black double arrow with the Chebyshev distance metric) with the nearest homology of neighbouring diagonals. If the maximum gap allowed for the identification of micro-rearrangements is at least 1, the isolated homology is identified as a mono-genic conserved segment.
(PDF)

S14 Fig Events simulated in MagSimus. (PDF)

S15 Fig Species tree with the numbers of events on each branch. (PDF)

S16 Fig Distribution of the length of inversions. The curve with the grey surface under the blue curve is the probability mass function (pmf) of the distribution of the lengths of reversed segments. The black line is the corresponding cumulated density function (cdf). The chosen function for the pmf is a discretisation of the gamma function with a shape parameter 0.1 and a scale parameter equal to 800 genes, truncated after 1330 genes. With this distribution, 53.9% of reversed segments are mono-genic, 57.7% of reversed segments contain one or two genes and 63.2% of the reversed segments have at most 5 genes.
(PDF)

S1 Text Description of the simulation with MagSimus. (DOCX)
