# Supplementary material for: High precision detection of conserved segments from synteny blocks
Source: PLoS One. 2017 Jul 3;12(7):e0180198. doi: 10.1371/journal.pone.0180198 (PMC5495381; doi:10.1371/journal.pone.0180198)
Supplement: S1 Fig — The arrow in the center, pointing towards the bottom, is the time arrow. The genome before and after each event is drawn to show how each event altered it. Colours and capital characters correspond to gene families. Genes outlined in black are ancestral genes except genes filled in white that are genes from families originating after the speciation (genes specific to one lineage). Genes not outlined in black are non-ancestral genes inserted due to duplication. Small characters after dots help here to differentiate copies and copied genes: A.a is not the same gene as gene A even if they are both in the family of gene A. Furthermore if gene A was once more duplicated there would be an instantiated copy named A.b newly inserted in the genome and if the gene A.a was duplicated there would be an instantiated copy named A.a.a. The dates of the events, that either include chromosomal rearrangements with breakpoints or ancestral gene deletions, are specified along the time arrow because they alter conserved segments. Chromosomal fusions, gene duplications and de novo gene births do not alter conserved segments. Corresponding evolutions of conserved segments along different lineages are drawn afterwards in S2 and S3 Figs. (PDF) [file pone.0180198.s001.pdf]

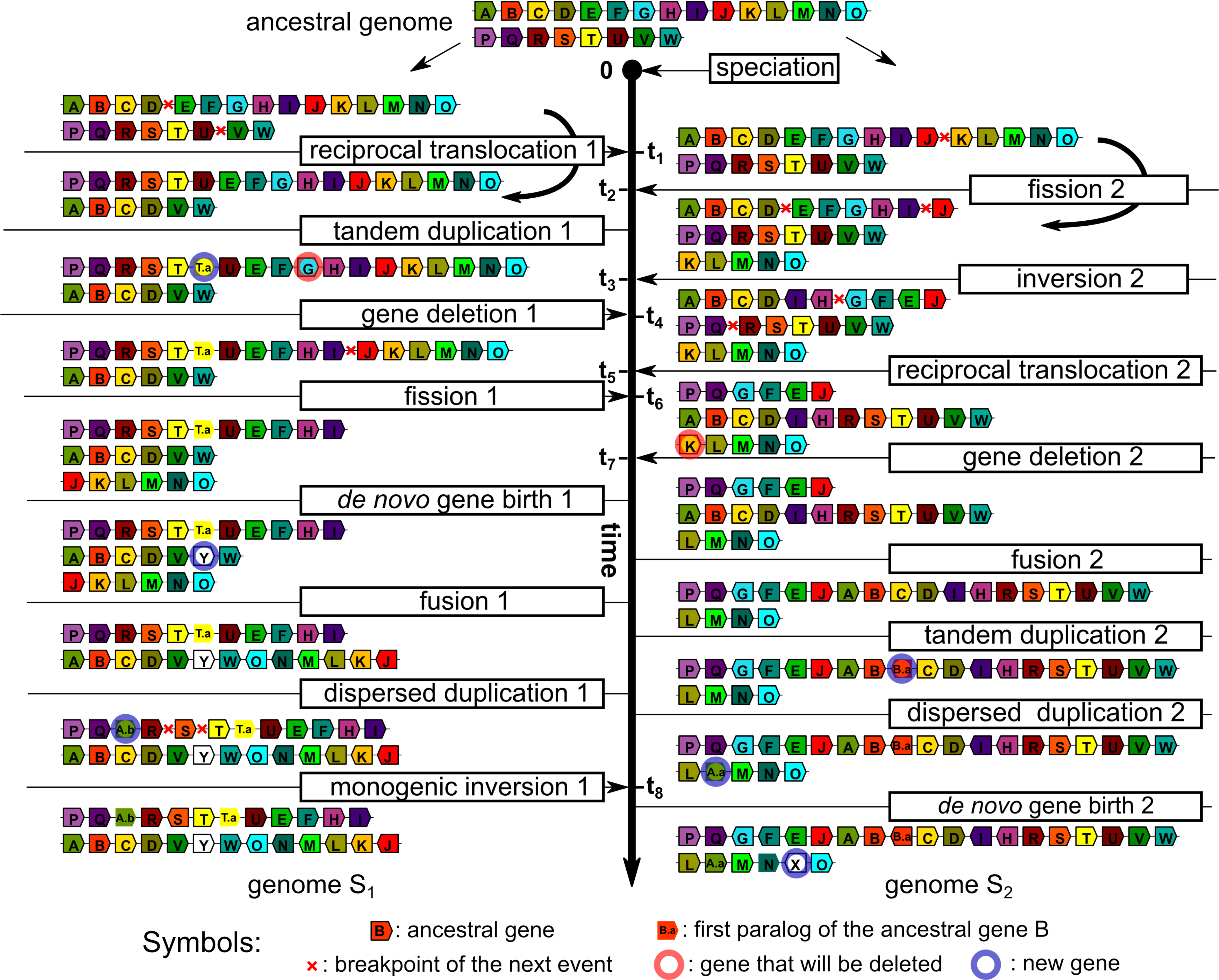

ancestral genome
speciation
0
reciprocal translocation 1
tandem duplication 1
gene deletion 1
fission 1
de novo gene birth 1
fusion 1
dispersed duplication 1
monogenic inversion 1
fission 2
inversion 2
reciprocal translocation 2
gene deletion 2
fusion 2
tandem duplication 2
dispersed duplication 2
de novo gene birth 2
t_1
t_2
t_3
t_4
t_5
t_6
t_7
t_8
time
genome S_1
genome S_2
Symbols:
B : ancestral gene
B.a : first paralog of the ancestral gene B
× : breakpoint of the next event
: gene that will be deleted
: new gene
