# Supplementary figures and images for: High precision detection of conserved segments from synteny blocks
Source: PLoS One. 2017 Jul 3;12(7):e0180198. doi: 10.1371/journal.pone.0180198 (PMC5495381; doi:10.1371/journal.pone.0180198)

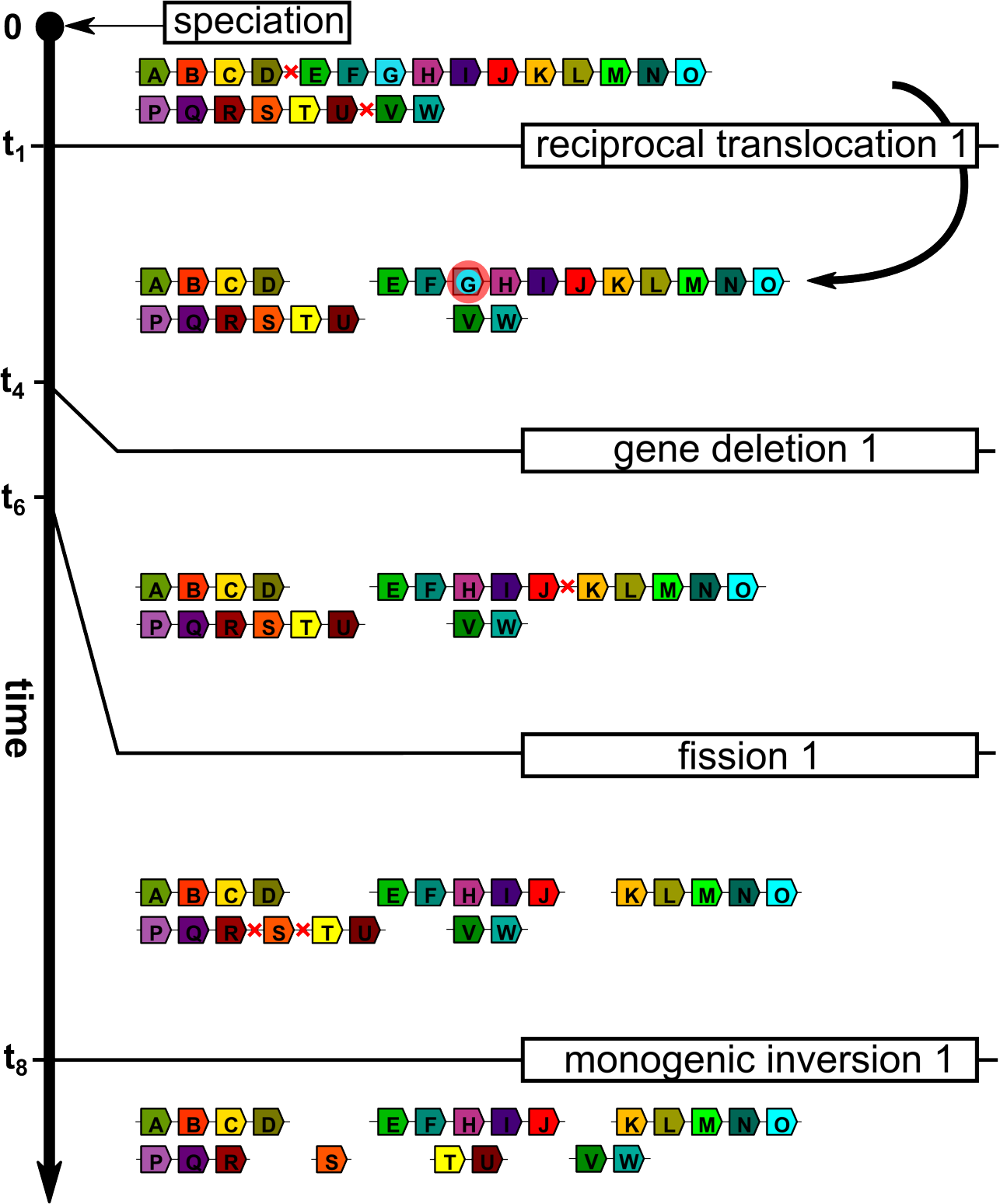

Supplement: S2 Fig — Between t = 0 and t = t1, before any alteration of the ancestral gene order or ancestral gene content, the conserved segments are exact copies of the ancestral chromosomes in the ancestral genome. The two breakpoints of the translocation 1 (in the first lineage) start breaking the conserved segments at the two corresponding spaces between ancestral genes. Afterwards, the gene deletion 1 removes the ancestral gene G from the conserved segments. Next, breakpoints associated with extremities of rearranged segments keep fragmenting conserved segments until the 7 final conserved segments from the ancestral genome to S1. (PDF) [file pone.0180198.s002.pdf]

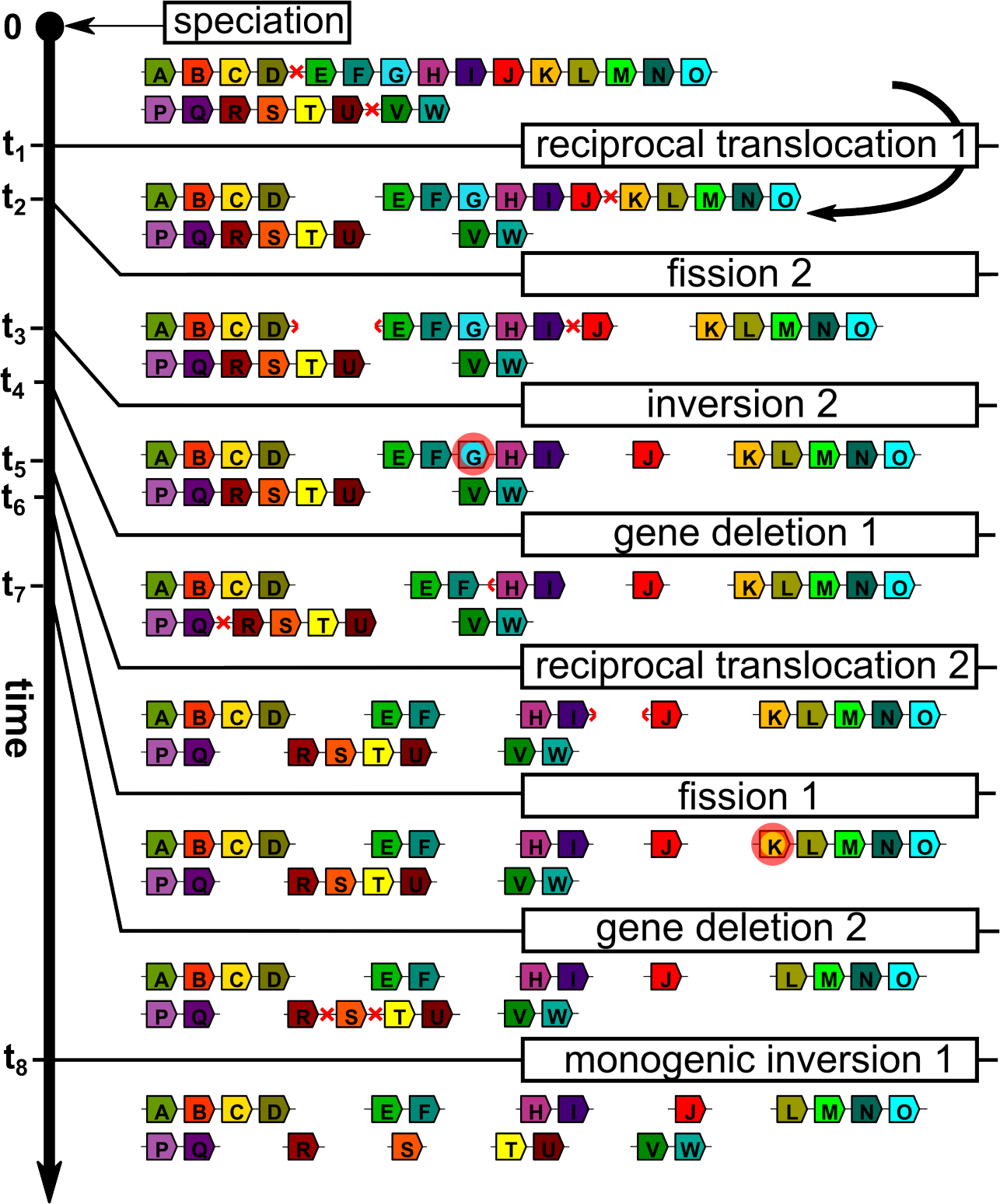

Supplement: S3 Fig — Here again, before the translocation 1 the conserved segments are exact copies of the ancestral chromosomes, and the translocation 1 (in the first lineage) starts breaking the conserved segments at two breakpoints. The next event altering conserved segments along both lineages is, in this case, an event in the second lineage: fission 2. Considering events in both lineages returns more and smaller conserved segments than in S2 Fig: here, when the evolution is finished, 10 segments of the ancestral genome have been conserved. In addition, more deletions of ancestral genes in conserved segments are expected when studying the conservation of ancestral segments in multiple lineages instead of in a unique lineage. For instance, the deletion of the ancestral gene G causes the loss of the ancestral gene G, in conserved segments from the ancestor to S1 and S2, even if the deletion only occurred in one lineage; same for the deletion of the ancestral gene F. (PDF) [file pone.0180198.s003.pdf]

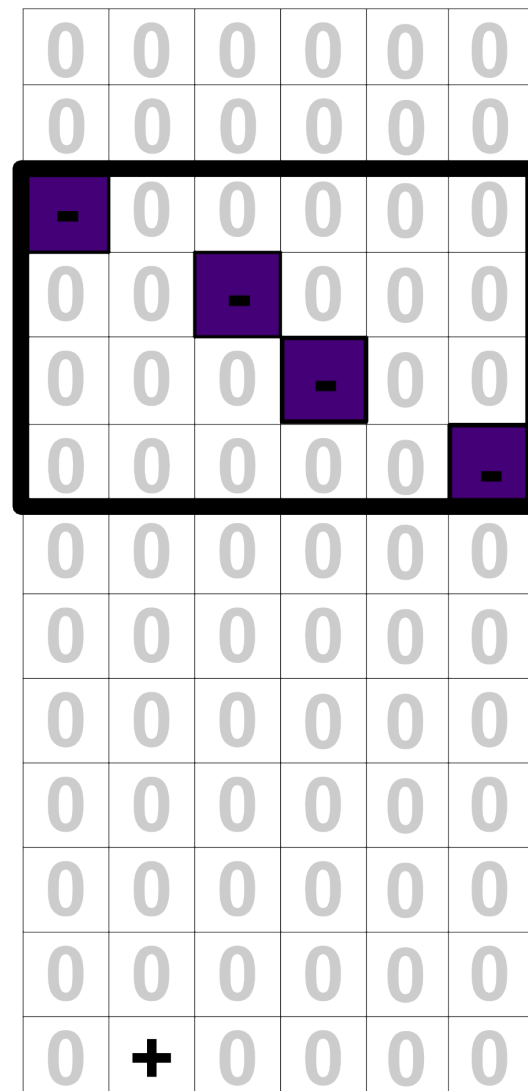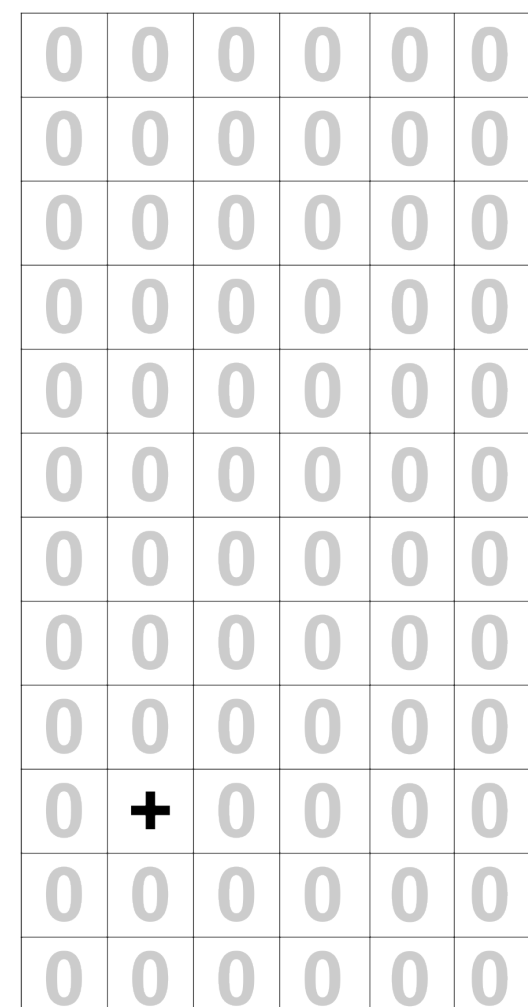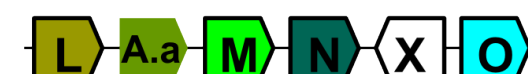

Supplement: S4 Fig — Each chromosome on the x-axis is ordered from left to right: it starts at left and ends at right. On the y-axis chromosomes are ordered from bottom to top. A gene has a positive orientation if its 3’-5’ orientation (its arrow here) points to the end of its host chromosome. In the contrary, it has a negative orientation if it points to the beginning of the chromosome. For instance, gene P of S1, gene P of S2 and gene E of S2 have positive orientations (they point either to the right or to the top) whereas gene E of S1 has a negative orientation, it points to the left. The matrix is an array of signs equal to +, − or 0. Non-0 signs correspond to homology signs. For instance, the homology sign corresponding to gene P in S1 and gene P in S2 is “+” because both genes have a positive orientation. In contrast, Gene E in S1 has a negative orientation and Gene E in S2 has a positive orientation thus the corresponding homology has a “-” sign. In the matrix, diagonals of conserved segments from the ancestor to S1 and S2 are outlined with black rectangles, and homologies within the same diagonal have the same colour. Both compared genomes have 2 chromosomes and the matrix is thus composed of 4 sub-matrices of homologies of the comparison of pairs of chromosomes. Remark: When the evolution from the ancestor to compared extant genomes is unknown, genes of extant genomes cannot be labelled with ancestral gene names and names of copies. Homology relationships between genes are usually estimated from comparisons of nucleotide sequences and, in practice, genes are labelled with family names. Consequently, ancestral gene localisations are often unknown and a localisation of an ancestral gene is often mixed among the locations of its copies. For instance, if we did not known the evolution depicted in S1 Fig, genes B and B.a would both be labelled with the name of their common family, B. Similarly, in Fig 4A of the main manuscript, two genes are labelled E because both genes are in the [file pone.0180198.s004.pdf]

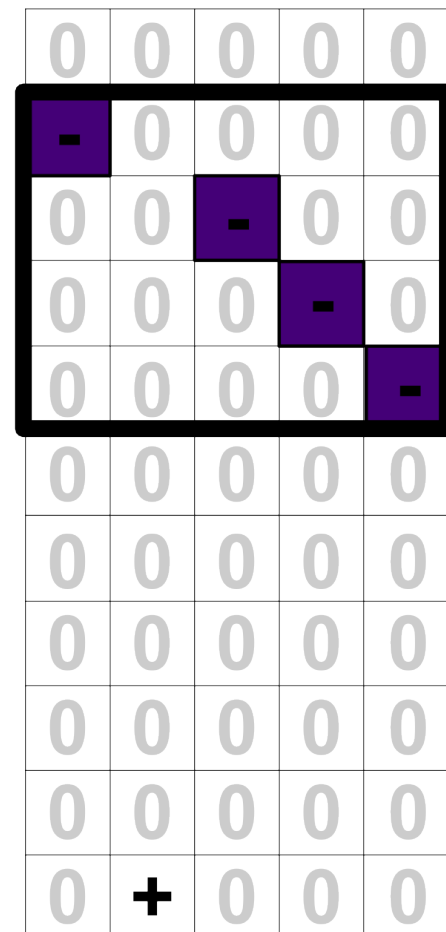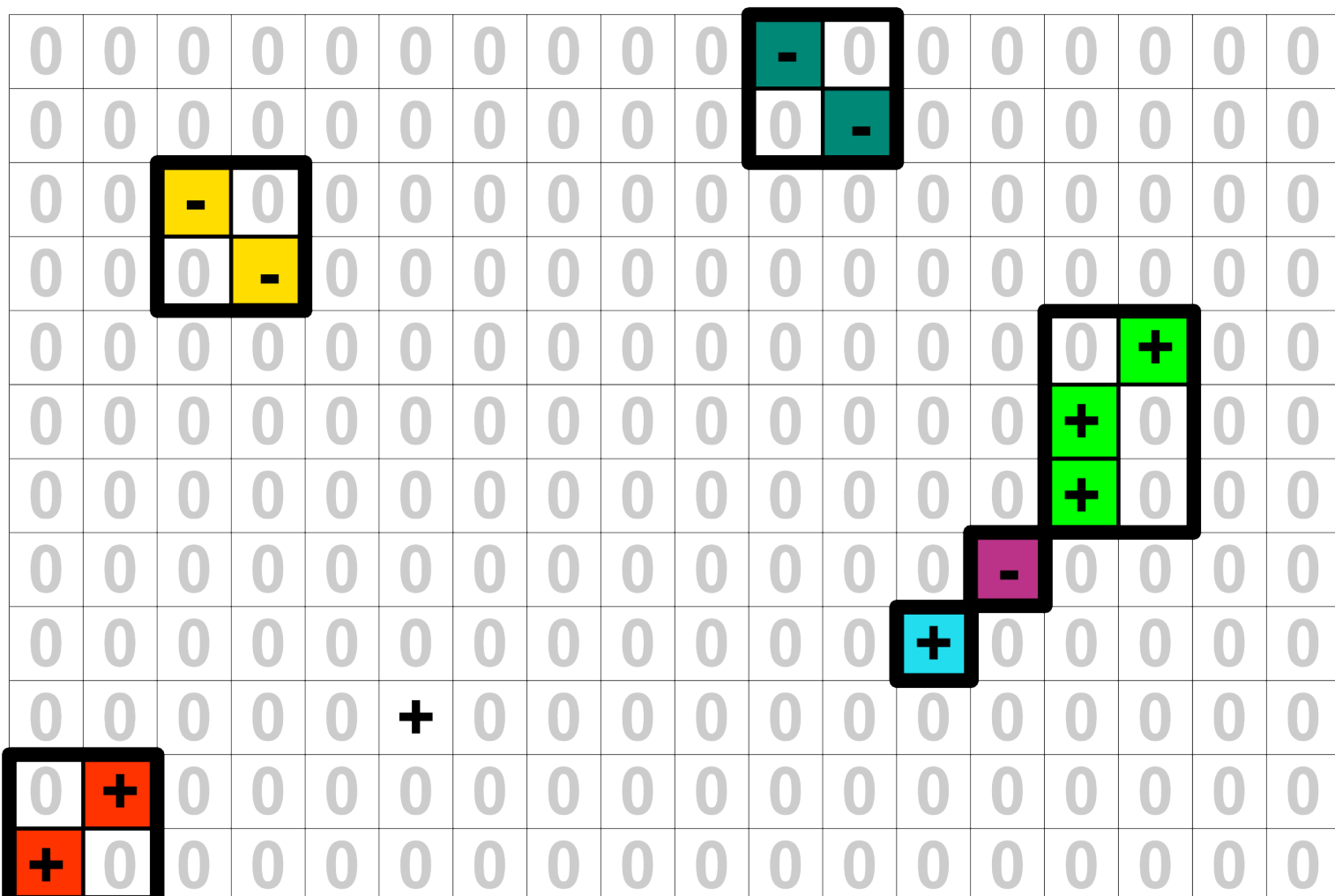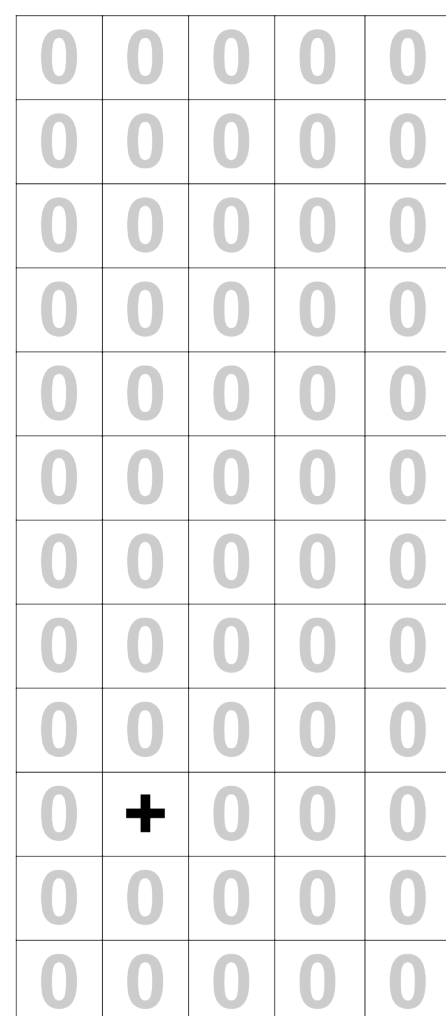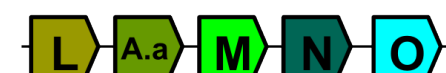

Supplement: S6 Fig — Keeping only genes with homologs in the compared genome removes artificial gaps due to de novo gene births and gene deletions but can neither solve artificial gaps due to dispersed duplications (A.a and A.b) nor packs of homologies caused by clusters of tandem duplicates (T and T.a or B and B.a). (PDF) [file pone.0180198.s006.pdf]

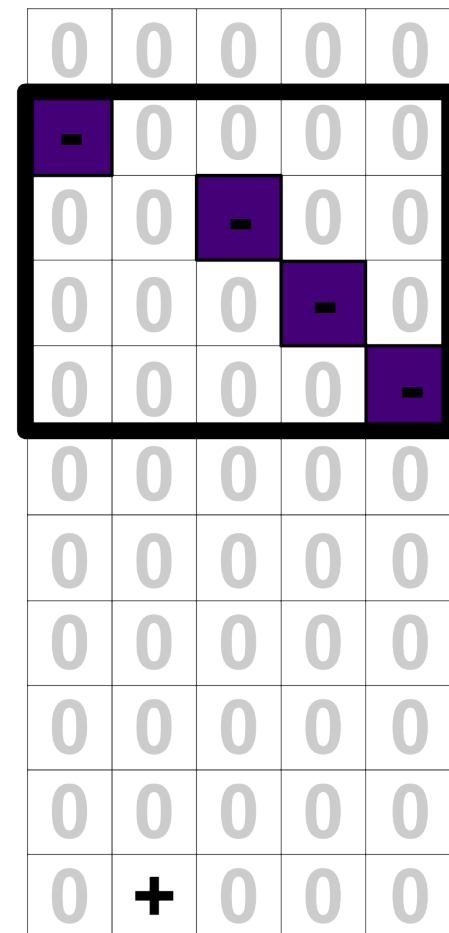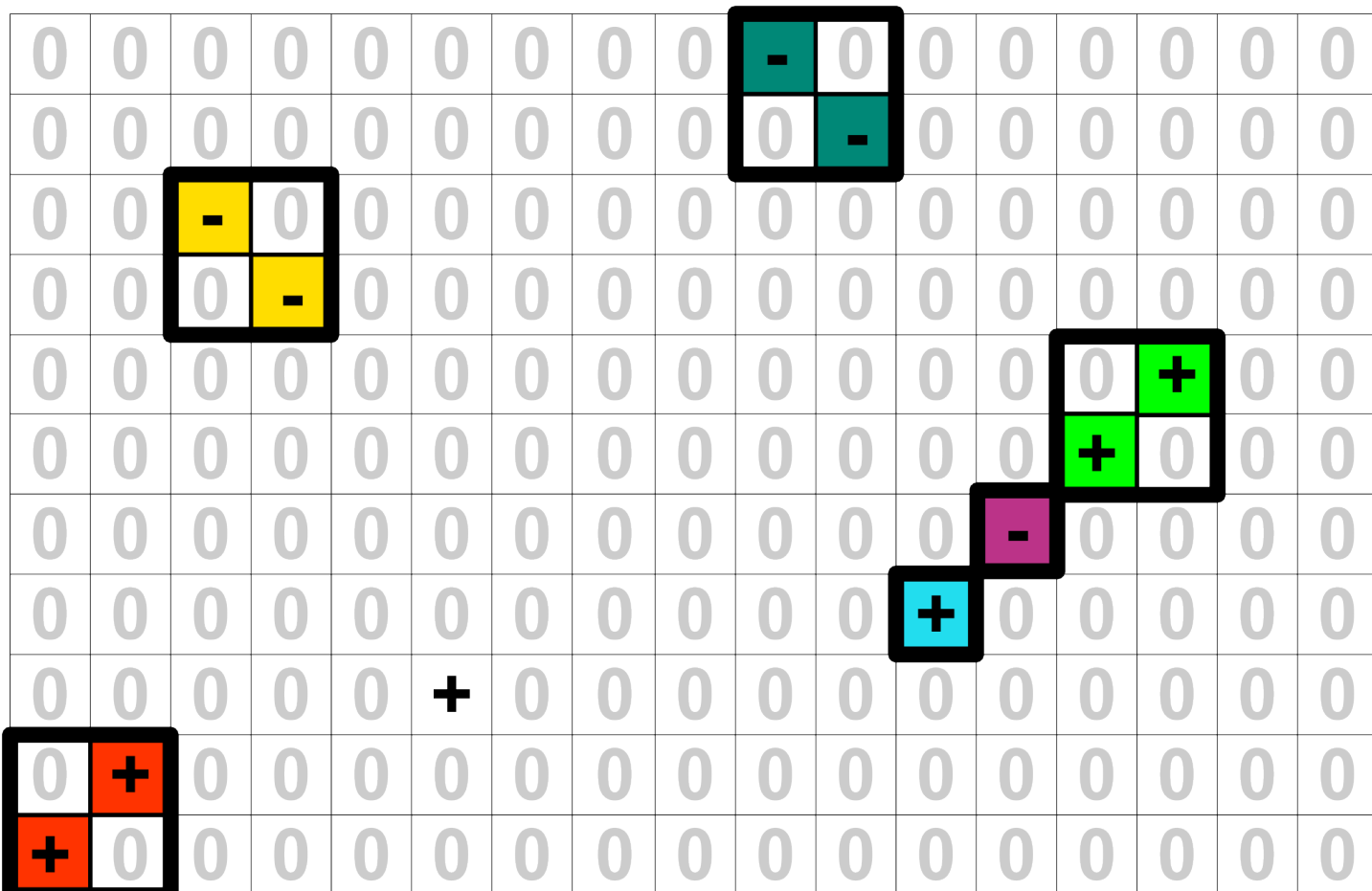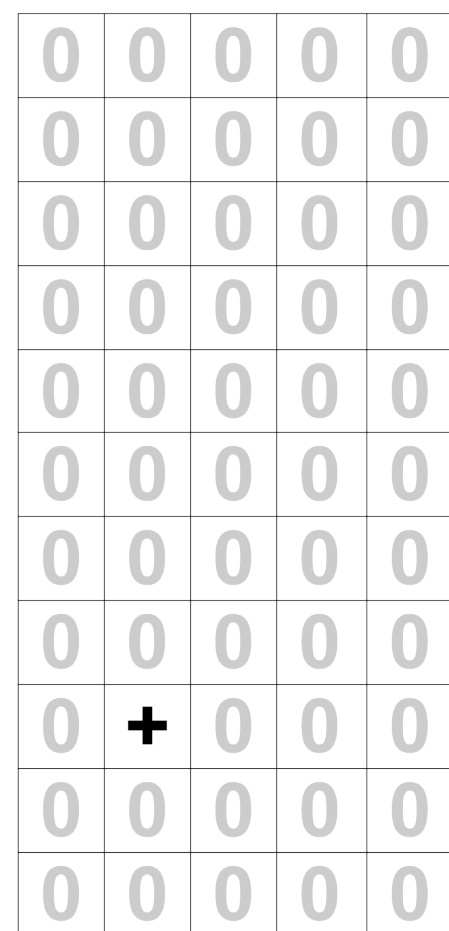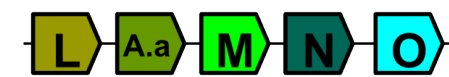

Supplement: S7 Fig — Collapsing clusters of tandem duplicates in addition to removing genes with no homolog in the other genome bring the matrix of homologies closer to the ideal matrix of homologies with perfectly filtered genomes (S5 Fig). Yet diagonals still contain artificial gaps caused by dispersed duplications, even with this intense filtering. Hence, in the absence of a better way to pre-process compared genomes, algorithms aiming at identifying synteny blocks or conserved segments, have to deal with artificial gaps; at least unitarian gaps scattered in genomes because of dispersed duplications. A gapMax = 0 won’t be sufficient to overcome these artificial gaps and values of gapMax at least equal to 1 are necessary. (PDF) [file pone.0180198.s007.pdf]

Gallus.gallus chr4:1-300

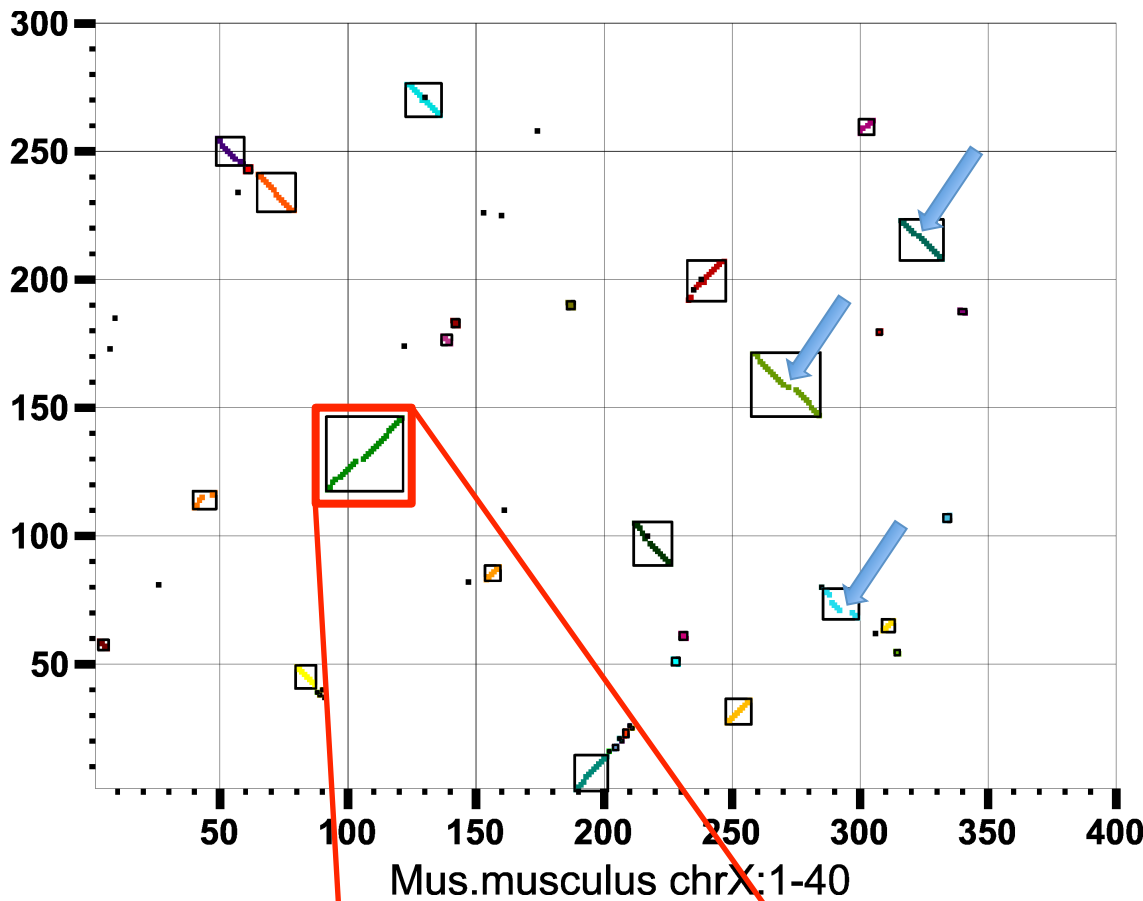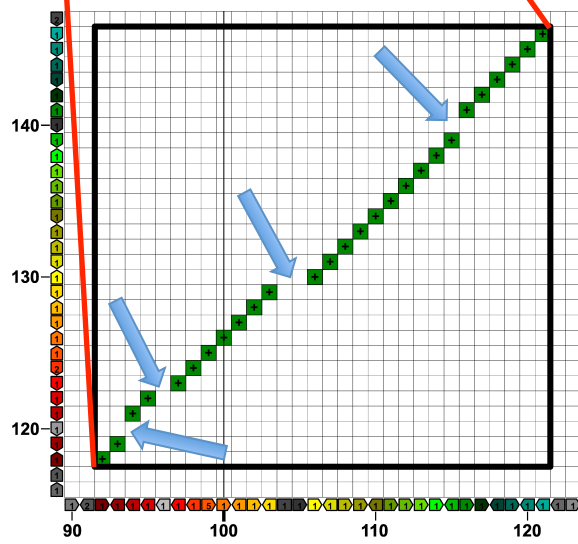

Supplement: S9 Fig — Both chromosomes contain only genes that have homologs in the other genome and clusters of genes duplicated in tandem have been collapsed. Blue arrows point towards examples of artificial gaps within diagonals of conserved segments. These artificial gaps seem to be due to dispersed duplications or errors (assembly errors, annotation errors or errors in gene families). A zoom of the region circled in red is also shown. (PDF) [file pone.0180198.s009.pdf]

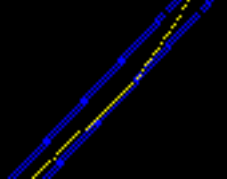

Supplement: S10 Fig — Yellow dots represent homologies in a base_cluster and blue dots represent the “confidence intervals” around the base_cluster, see the documentation of i-ADHoRe 3.0 [6]. Here also, after the pre-processing of i-ADHoRe 3.0 (filter + collapse of clusters of tandem duplicates), artificial gaps remain within a diagonal of a conserved segment. (PDF) [file pone.0180198.s010.pdf]

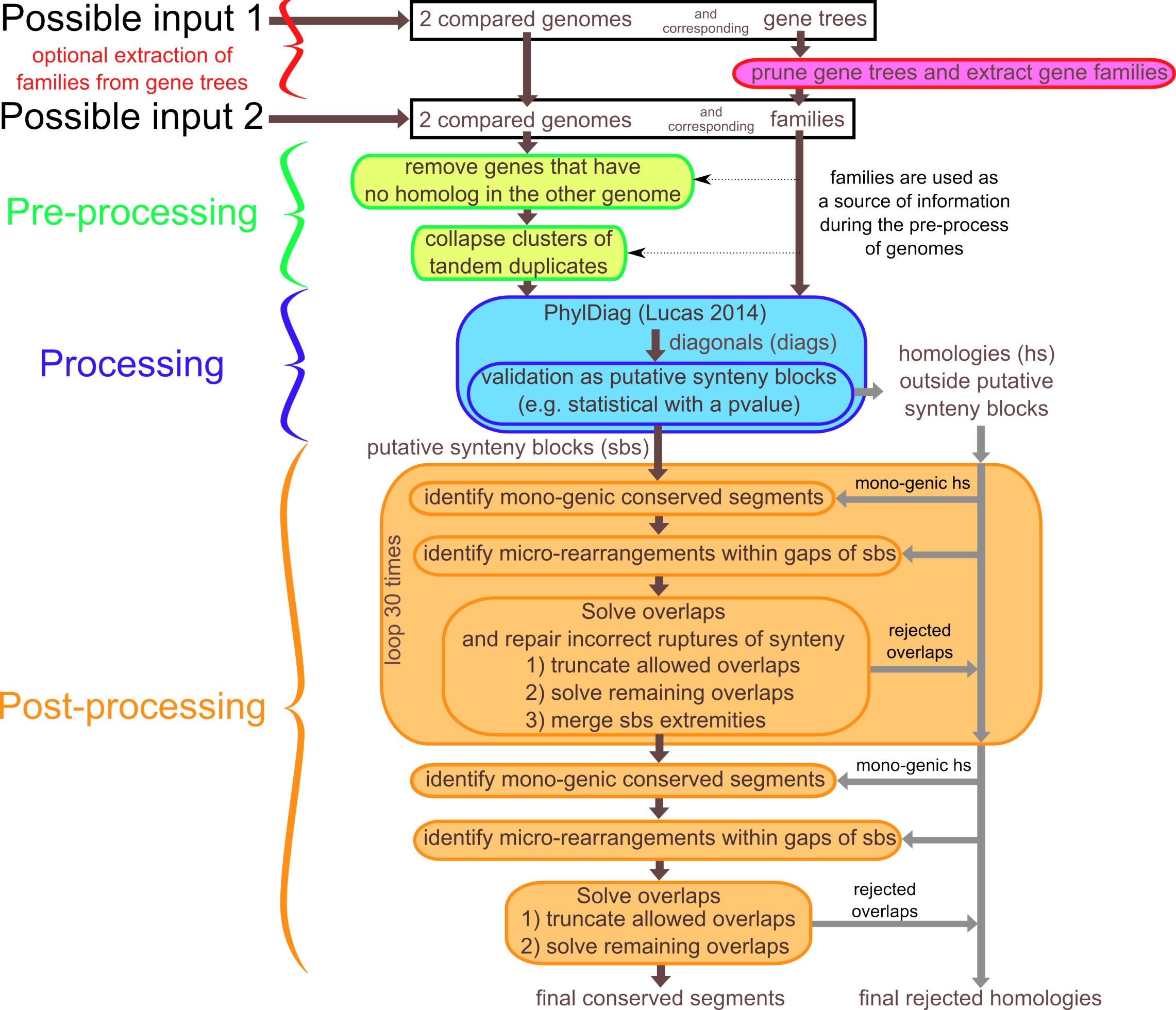

Supplement: S11 Fig — The scripts/ folder, in the LibsDyogen deposit, provides tools to prune.nhx gene trees and extract gene families from pruned gene trees. The new version of PhylDiag (from v2.0.0-alpha) includes the pre-processing and post-processing steps (https://github.com/DyogenIBENS/PhylDiag). (PDF) [file pone.0180198.s011.pdf]

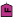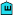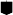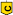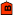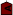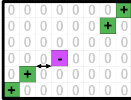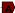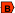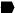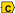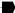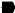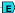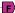

Supplement: S12 Fig — An isolated homology is distant with a maximum gap of 1 (length of the black double arrow with the Chebyshev Distance Metric) with the nearest homology of its surrounding diagonal. If the maximum gap allowed for the identification of micro-rearrangements is at least 1, the isolated homology is identified as a mono-genic conserved segment. Black genes are probable ancestral genes inserted because of dispersed duplications or they might be due to errors in data (annotation errors or errors in families). (PDF) [file pone.0180198.s012.pdf]

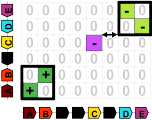

Supplement: S13 Fig — An isolated homology is distant with a maximum gap of 1 (length of the black double arrow with the Chebyshev distance metric) with the nearest homology of neighbouring diagonals. If the maximum gap allowed for the identification of micro-rearrangements is at least 1, the isolated homology is identified as a mono-genic conserved segment. (PDF) [file pone.0180198.s013.pdf]

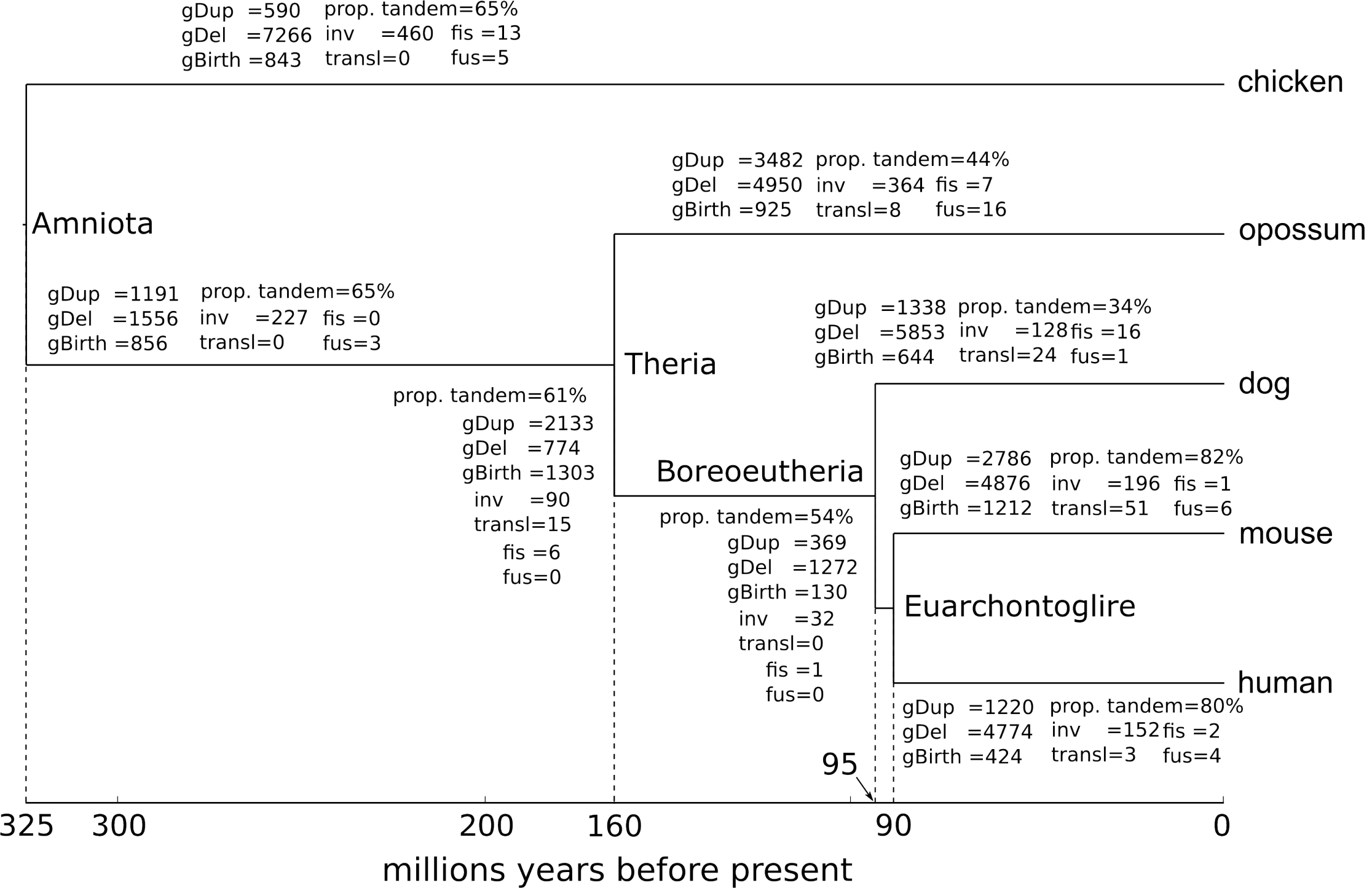

Supplement: S15 Fig — (PDF) [file pone.0180198.s015.pdf]

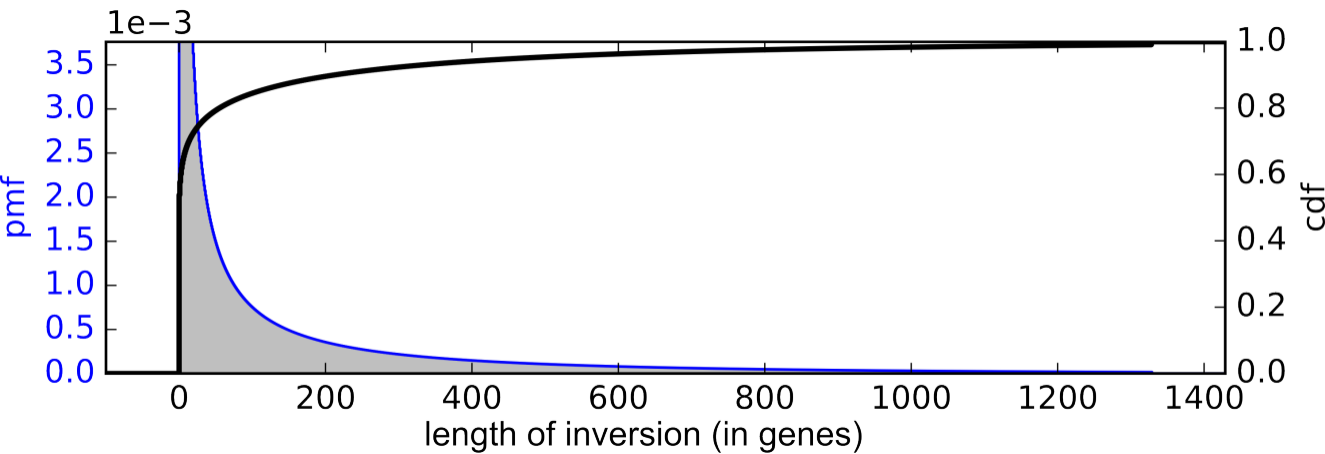

Supplement: S16 Fig — The curve with the grey surface under the blue curve is the probability mass function (pmf) of the distribution of the lengths of reversed segments. The black line is the corresponding cumulated density function (cdf). The chosen function for the pmf is a discretisation of the gamma function with a shape parameter 0.1 and a scale parameter equal to 800 genes, truncated after 1330 genes. With this distribution, 53.9% of reversed segments are mono-genic, 57.7% of reversed segments contain one or two genes and 63.2% of the reversed segments have at most 5 genes. (PDF) [file pone.0180198.s016.pdf]
