# Supplementary material for: High precision detection of conserved segments from synteny blocks
Source: PLoS One. 2017 Jul 3;12(7):e0180198. doi: 10.1371/journal.pone.0180198 (PMC5495381; doi:10.1371/journal.pone.0180198)
Supplement: S5 Fig — In this matrix, conserved segments from the ancestor to the two compared extant genomes can be identified as perfect diagonals with no gaps in the matrix of homologies. Remark: When compared genomes are not filtered (S4 Fig), diagonals of conserved segments are blurred with artificial gaps. Dispersed duplications (A.a and A.b), de novo gene births (X and Y) and ancestral gene deletions (G and K) create artificial gaps within diagonals (or around diagonals) of conserved segments that would not exist if genomes were perfectly filtered (S5 Fig). Furthermore if compared genomes are not filtered, diagonals of conserved segments are also blurred with artificial packs of homologies, vertical or horizontal (S5 Fig) or even rectangular (see later in S8 Fig) caused by clusters of tandem duplicates. S6 and S7 Figs will give examples of matrices of homologies that are obtained with two pre-processings of compared genomes that gradually approach the perfect filtering. (PDF) [file pone.0180198.s005.pdf]

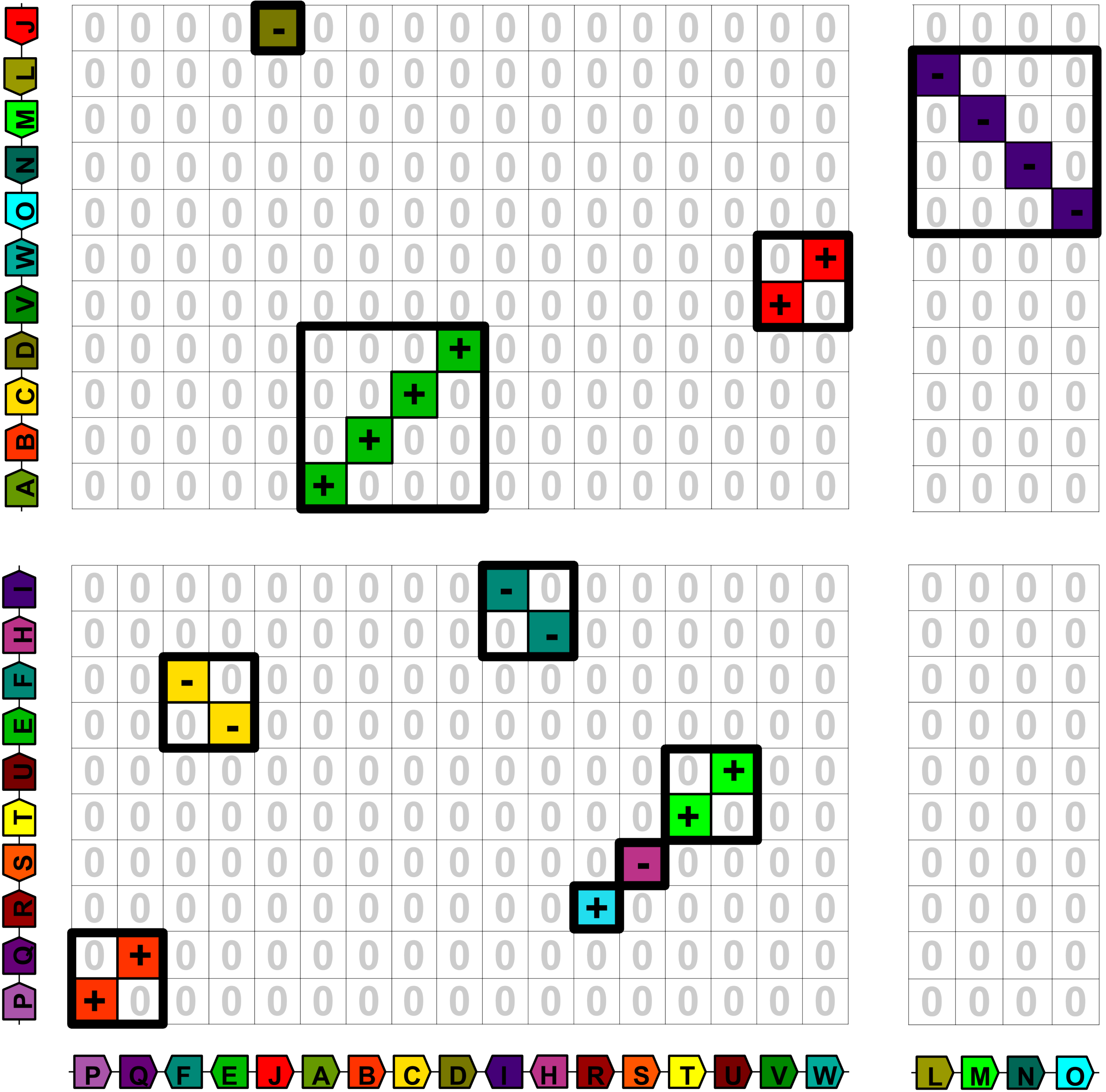
P Q F E J A B C D I H R S T U V W
L M N O
