# Supplementary material for: High precision detection of conserved segments from synteny blocks
Source: PLoS One. 2017 Jul 3;12(7):e0180198. doi: 10.1371/journal.pone.0180198 (PMC5495381; doi:10.1371/journal.pone.0180198)
Supplement: S8 Fig — An ancestral chromosome of three genes ABC evolves with 2 different scenarios until 2 extant species S1 and S2. In the first scenario, left column, the gene B is duplicated in tandem, before the speciation, i.e. the duplication happens between the initial genome and the most recent common ancestor of S1 and S2, MRCA(S1, S2). The conserved segment from the MRCA to S1 and S2 is thus made of 4 ancestral genes. Pruning the gene tree of gene B at the level of the MRCA divides the family of gene B into two families: a smaller family of gene B and a new family corresponding to the legacy of gene B.a. The differentiation of families of genes B and B.a makes it possible to identify the true 4 ancestral genes in the segment conserved from the MRCA to S1 and S2. The second scenario, right column, has two duplications after the speciation that give rise to a similar configuration of tandem duplications in extant species. Contrary to the previous scenario, here, both duplications in tandem happen after the MRCA and insert non-ancestral copies of the gene B: B.a and B.b. There are thus only three genes, ABC, in the conserved segment from the MRCA to S1 and S2. Pruning the gene tree of gene B does not change the family of gene B in this case since at the level of the MRCA only gene B exists. Thus tandem duplicates are visible in the matrix of homologies and collapsing clusters of tandem duplicates leads once more to the true number of 3 ancestral genes in the detected segment conserved from the MRCA to S1 and S2. If the scenario of the right column happened, and if the phylogenetic algorithm used for inferring gene trees made a mistake, and found the gene tree of the left column, our detection of conserved segments, as diagonals in the matrix of homologies would make an error. It would detect 4 ancestral genes in the segment conserved from the MRCA to S1 and S2 instead of the 3 true ancestral genes. Such interdependences between gene trees and collinearity have been used to improv [file pone.0180198.s008.pdf]

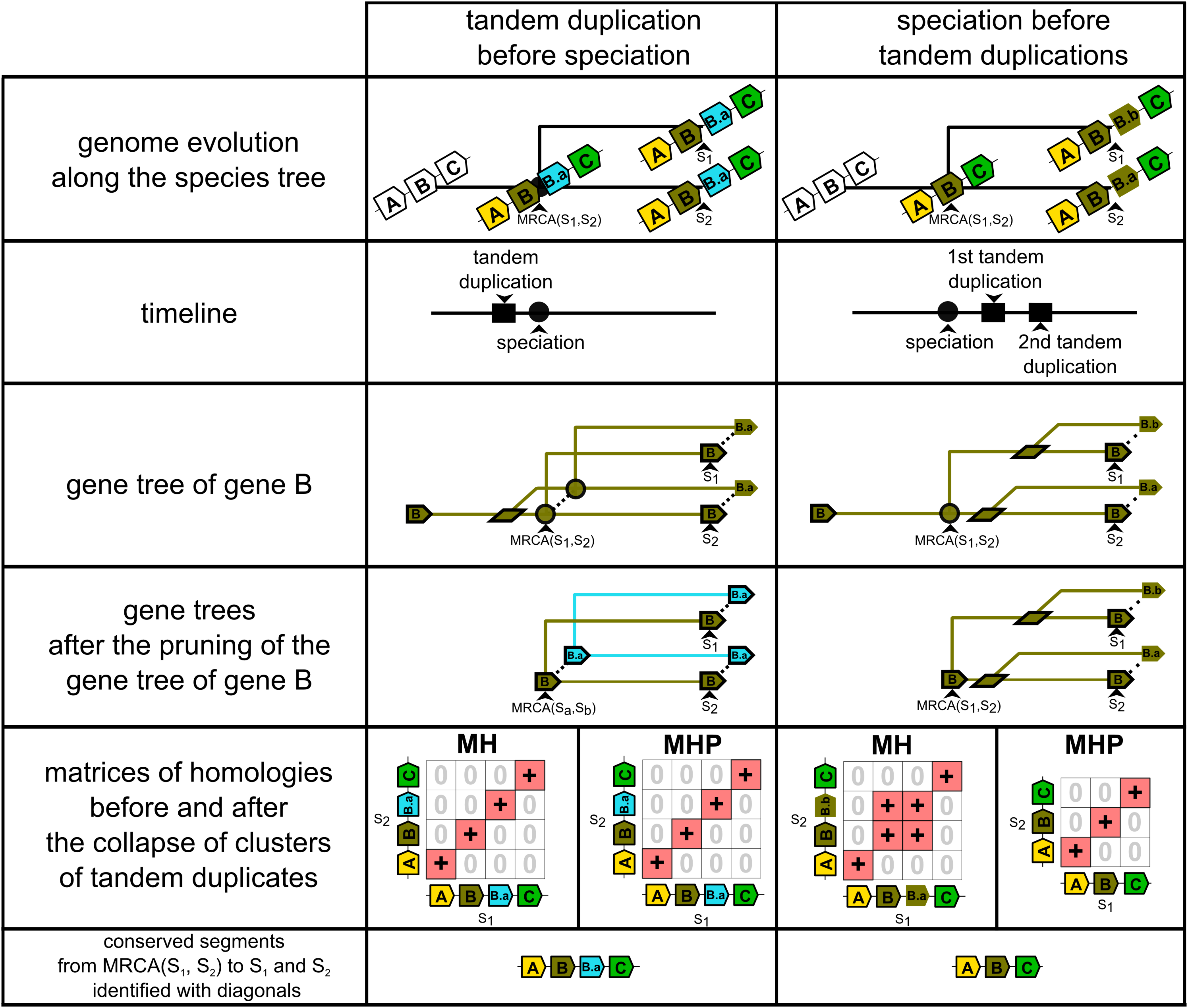
tandem duplication before speciation
speciation before tandem duplications
genome evolution along the species tree
A B C
A B B.a C
MRCA(S1,S2)
S1
S2
A B B.b C
A B B.a C
timeline
tandem duplication
speciation
1st tandem duplication
2nd tandem duplication
gene tree of gene B
B
B.a
B.b
gene trees after the pruning of the gene tree of gene B
MRCA(Sa,Sb)
matrices of homologies before and after the collapse of clusters of tandem duplicates
MH
MHP
0
+
conserved segments from MRCA(S1, S2) to S1 and S2 identified with diagonals
