# Supplementary material for: High precision detection of conserved segments from synteny blocks
Source: PLoS One. 2017 Jul 3;12(7):e0180198. doi: 10.1371/journal.pone.0180198 (PMC5495381; doi:10.1371/journal.pone.0180198)
Supplement: S14 Fig — (PDF) [file pone.0180198.s014.pdf]

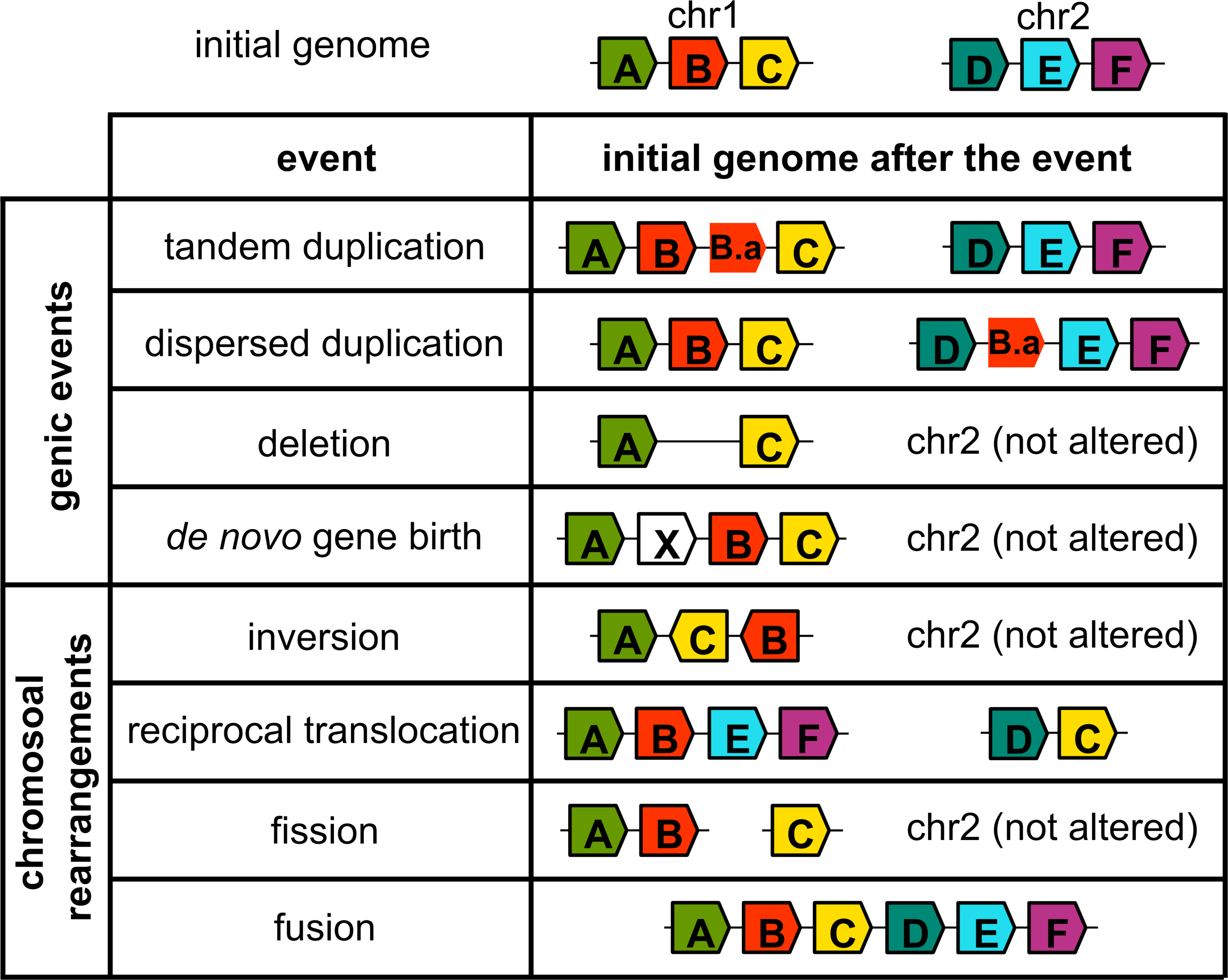
initial genome
chr1
A B C
chr2
D E F
event
initial genome after the event
genic events
tandem duplication
A B B.a C
D E F
dispersed duplication
A B C
D B.a E F
deletion
A C
chr2 (not altered)
de novo gene birth
A X B C
chr2 (not altered)
chromosoal rearrangements
inversion
A C B
chr2 (not altered)
reciprocal translocation
A B E F
D C
fission
A B
C
chr2 (not altered)
fusion
A B C D E F
