# Supplementary material for: High precision detection of conserved segments from synteny blocks
Source: PLoS One. 2017 Jul 3;12(7):e0180198. doi: 10.1371/journal.pone.0180198 (PMC5495381; doi:10.1371/journal.pone.0180198)
Supplement: S1 Text — (DOCX) [file pone.0180198.s017.docx]

**S1 Text. Description of the simulation with MagSimus.**

Magsimus (Modeling Ancestral Genomes By Simulations) is a simulator that designs an initial ancestral genome and makes it evolve *in silico* within a species tree. When the simulation starts, the initial genome is copied and the simulator recursively and independently evolves the copies by simulating events, on both branches arising from the root of the species tree. At the end of each branch the genome of the intermediary ancestor is recorded. This process is recursive at each speciation node and stops at the leaves of the tree. At the end of the simulation, the genomes at the leaves are the simulated extant genomes and they are also recorded. Given a set of real extant genomes, MagSimus aims at simulating a set of simulated extant genomes that reproduces as much as possible the features of the real set. All simulated events altering genomes during the simulated evolution are shown in S14 Fig.

Inputs of MagSimus are:

- features of the initial ancestral genome:
  - number of chromosomes
  - number of genes
  - number of genes in each chromosome
- the species tree of the simulated evolution (the initial ancestor is at the root of the tree, intermediary ancestors are at the other internal nodes of the tree and extant species are at the leaves of the tree)
- parameters of evolution:
  - the distribution of the length of reversed segments
  - samplings of rearranged chromosomes
  - numbers of each event on each branch

Ouputs of MagSimus are:

- the initial ancestral genome
- intermediary ancestral genomes
- extant genomes
- sets of gene families.
- conserved segments recorded during the simulation, similarly to S1, S2 and S3 Figs

A set of gene families is returned for each initial or intermediary ancestor. Each family of a set gathers all genes deriving from one unique gene of the corresponding ancestor, as if families were built from gene trees pruned at the level of this ancestor.

**Overview of the functioning of MagSimus**

The order of events is randomly chosen at the beginning of each branch. During simulation, conserved segments along each branch are recorded. After the simulation, conserved segments from each ancestor to all pairs of extant descendants are computed based on the segments conserved on each branches [1]. When a rearrangement event occurs, depending on the type of rearrangement, the chromosome(s) involved is(are) sampled either proportionally to its(their) length(s) (a chromosome twice as long as another chromosome has twice more chance of being rearranged) or uniformly (all chromosomes have the same chance of being chosen).

**Parameterisation**

We simulated the evolution from the Amniota genome to 5 extant species: human, mouse, dog, opossum and chicken. The design of the ancestral genome and the evolutionary parameters have been chosen in order to simulate extant genomes that reproduce features of the real extant genomes, downloaded in Ensembl database v81.

We designed an initial ancestral genome of Amniota with 21 chromosomes and 19547 ancestral genes, a number of genes predicted from gene trees of Ensembl v81. The number of chromosomes in Amniota has been computed using ChromEvol [2] on an expanded set of 21 vertebrates, with known numbers of chromosomes, closely related to our five species and including outgroups [1]. The number of genes in each chromosome corresponds to a distribution that averages the distributions of the length of chromosome (in genes) in the five simulated extant genomes. The computation of the average distribution is explained in more details in [1].

The species tree used (S15 Fig) has been downloaded from the Ensembl database [3] (especially the topology and speciation dates) and the numbers of genic events (gene duplications, gene deletions and *de novo* gene births) on each branch are estimated from gene trees of Ensembl v81 [3].

The sampling of chromosomes for inversions is proportional to chromosome lengths. Thus the global density of inversion breakpoints (#breakpoints of inversions on a chromosome/length of the chromosome in genes) is the same in small and long chromosomes. Whatever our choice of chromosome sampling for translocations, fissions and fusions (either uniform or proportional to chromosome length) we observed an exponential distribution of chromosome length in extant simulated genomes: small chromosomes are too small and long chromosomes are too long compared to real chromosomes. Thus we chose the chromosome samplings of translocations, fissions and fusions in such a way as to lessen the unrealistic exponential distribution of simulated chromosome length. In practice, samplings selected tend to increase the lengths of small chromosomes and decrease lengths of long chromosomes: chromosomes involved in a translocation are chosen uniformly, chromosomes broken by fissions are chosen proportionally to lengths and chromosomes involved in fusions are chosen uniformly.

The numbers of fissions and fusions along branches of the species tree have been computed from the results of ChromEvol on the set of 21 vertebrates presented above. From the estimated numbers of chromosomes, at each internal node of the species tree of the 21 extant vertebrates, we estimated the numbers of fissions and fusions in the species tree of our five species, by parsimony. We also estimated the numbers of inversions and translocations between each pair of real genomes of the Ensembl database. We used for that a previously published estimator [4], and segments of PhylDiag [5] conserved between pairs of extant species. With the estimated numbers of inversions we built a distance matrix with numbers of inversions in place of distances. The numbers of inversions on the branches of the species tree were calculated with this matrix and the method of Non-Negative Least Squares (NNLS) [6][7]. More precisely we used the python function nnls of the scipy.optimize package [8]. The number of translocations on each branch of the species tree was computed similarly. Finally, an optimisation on the numbers of simulated translocations and inversions was performed until we obtained estimations close to the estimations from real data. In brief, we launched a large number of simulations, with gradually updated numbers of simulated inversions and translocations, until the numbers of inversions and translocations estimated from simulated data converged to the numbers of inversions and translocations estimated from real data.

The length of segments to reverse is randomly chosen, from a gamma probability distribution, with a shape parameter 0.1 and a scale parameter equal to 800 genes, truncated after 1330 genes (S16 Fig). This distribution of the length of inversions was computed after an optimisation process, over the two parameters of the gamma distribution.

The criterion of convergence was here the minimisation of an overall *error of realism*. The overall error of realism of our parameterisations has been computed by integrating 4 criteria:

- the number of chromosomes of each extant genome ($value, v=c^{i})$
- the distribution of chromosome length of each extant genome ($distribution, d=\gamma^{i})$
- the number of conserved segments detected between each pair of extant genomes ($value, v=b^{j,k})$
- the distribution of the length of segments conserved between each pair of extant genomes ($distribution, d=\beta^{j,k})$

With $i$, the index of an extant genome, $i\in\left[ 1,5 \right];$ and $\left( j,k \right)\in C_{5}^{2},$ one of the $\binom{5}{2}$ combinations of two extant genomes, with $C_{5}^{2}$ the set of all combinations of two genomes among five.

When a simulated scalar value $sv$ is compared to a real scalar value $rv$ (e.g. the simulated and real numbers of chromosomes, ${sc}^{i}$ and ${rc}^{i}$), the error of realism is the ratio

$$\rho\left( sv,rv \right)=\frac{sv}{rv}.$$

When a simulated distribution $sd$ is compared to a real distribution $rd$ (e.g. the simulated and real distributions of the length of chromosomes, ${s\gamma}^{i}$ and ${r\gamma}^{i}$), the error of realism is the ratio

$$\rho\left( sd, rd \right)=\frac{csd\left( W \right)}{crd\left( W \right)},$$

with $csd$, the cumulated simulated distribution, $crd$ the cumulated real distribution,

$$W= \underset{a}{\mathrm{argmax}} \left\langle\frac{csd(a)}{crd(a)} \right\rangle$$

and the function $\left\langle. \right\rangle:x \to\left\langle x \right\rangle=\left\{ \begin{matrix} x & if & 1\leq x \\ \frac{1}{x} & else & \end{matrix} \right.$.

Intuitively, $\rho\left( sd, rd \right)=\frac{csd\left( W \right)}{crd\left( W \right)}$ can be understood as a “ratio version” of the Kolmogorov-Smirnov statistic, commonly used to compare distributions; the “ratio version” uses here a ratio for the comparison instead of an arithmetic subtraction.

For each criterion and for each extant species, or each pair of extant species, the geometrical means of the errors of a parameterisation are computed over 100 simulations. For instance:

- $\rho\left( c^{i} \right)= \sqrt[100]{\prod_{n=1}^{100} \rho_{n}}$, with $\rho_{n}=\rho\left( sc_{n}^{i},{rc}^{i} \right)=\frac{sc_{n}^{i}}{{rc}^{i}}$, the error of realism the n^th^ simulation, when comparing the number of chromosomes.
- $\rho\left( \beta^{j,k} \right)= \sqrt[100]{\prod_{n=1}^{100} \rho_{n}}$, with here $\rho_{n}=\rho\left( s\beta_{n}^{j,k},r\beta^{j,k} \right)=\frac{s\beta_{n}^{j,k}}{r\beta^{j,k}}$, the error of the n^th^ simulation, when comparing distributions of the length of conserved segments.

These errors of realism of a parameterisation are edited to ensure that they are all higher than 1, using the function $\left\langle. \right\rangle$. Finally, once more, we computed the geometrical averages over the 5 extant species and the $\binom{5}{2}=10$ pairs of extant species. For instance:

- $\rho_{c}=\sqrt[5]{\prod_{i=1}^{5} \left\langle\rho\left( c^{i} \right) \right\rangle}$
- $\rho_{\beta}=\sqrt[10]{\prod_{(j,k)\in C_{5}^{2}} \left\langle\rho\left( \beta^{j,k} \right) \right\rangle}$

With our parameterisation, geometrical and general errors of realism are:

- $\rho_{c}=1.01$, meaning that, on average, the number of chromosomes in simulated extant genomes is distant by a factor 1.01 from the real number of chromosomes in real extant genomes.
- $\rho_{\gamma}=4.14$, meaning (roughly) that, on average, the number of the smallest simulated chromosomes in extant genomes is distant by a factor 4.14 from the number of the smallest chromosomes in real extant genomes. In practice we know that the lengths of the smallest simulated extant chromosomes are too small thus we can (roughly) expect that they are 4.14 times too many small chromosomes in simulations compared to reality. Other studies also mention that the distribution of chromosome length is difficult to reproduce [9,10].
- $\rho_{b}=1.01$, meaning that, on average, the number of conserved segments in pairwise comparisons of simulated extant genomes is distant by a factor 1.01 from the real number of conserved segments in pairwise comparisons of real extant genomes.
- $\rho_{\beta}=1.27$, meaning (still roughly) that, on average, the number of the smallest conserved segments in pairwise comparisons of simulated extant genomes is distant by a factor 1.27 (either over or under) from the number of the smallest conserved segments in pairwise comparisons of real extant genomes.

A null hypothesis of using a uniform distribution of inverted segment length, instead of the gamma distribution, with all other parameters identical, returns unchanged $\rho_{c}=1.01$ and $\rho_{b}=1.01$, a rather similar $\rho_{\gamma}=3.84$ and a very different $\rho_{\beta}=8.00$.

In [1] we discussed the limits of our simulator to reproduce breakpoint reuses and fragile regions [11], that may be an important phenomenon in real data, and how we plan to quantify and integrate this phenomenon in MagSimus and in the calculation of the error of realism.

Inversions outnumber other rearrangements, and 63.2% of the reversed segments have at most 5 genes, thus the number of inversions on each branch, multiplied by 63.2%, can be a rough approximation of the numbers of micro-rearrangements on each branch. However there is no convention on the maximal length of a micro-rearrangement. The value of the *gapMax* parameter, arbitrarily fixed by users when they detect synteny blocks, is usually taken as the threshold between micro- and macro-rearrangements.

More details on the calculation of the error of realism and the simulator can be found in [1]. A set of simulated genomes, gene families and conserved segments corresponding to the same simulation can be downloaded in the GitHub deposit of PhylDiag <https://github.com/DyogenIBENS/PhylDiag/tree/master/data/benchmark>.

**References**

1. Lucas JM. Etude de l’évolution de l’ordre des gènes de vertébrés par simulation [Internet]. 2016. Available: tel.archives-ouvertes.fr/tel-01398369/document

2. Glick L, Mayrose I. ChromEvol: Assessing the Pattern of Chromosome Number Evolution and the Inference of Polyploidy along a Phylogeny. Mol Biol Evol. 2014;31: 1914–22. doi:10.1093/molbev/msu122

3. Cunningham F, Amode MR, Barrell D, Beal K, Billis K, Brent S, et al. Ensembl 2015. Nucleic Acids Res. 2015;43: 662–669. doi:10.1093/nar/gku1010

4. Mazowita M, Haque L, Sankoff D. Stability of rearrangement measures in the comparison of genome sequences. J Comput Biol. 2006;13: 554–566. doi:10.1089/cmb.2006.13.554

5. Lucas JM, Muffato M, Crollius HR. PhylDiag : identifying complex synteny blocks that include tandem duplications using phylogenetic gene trees. BMC Bioinformatics. 2014;15: 1–15. doi:10.1186/1471-2105-15-268

6. Lawson C, Hanson R. Solving Least Squares Problems. Society for Industrial and Applied Mathematics; 1995. doi:10.1137/1.9781611971217

7. Felsenstein J. Inferring phylogenies. Sinauer Associates; 2004.

8. Jones E, Oliphant T, Peterson P, others. SciPy: Open source scientific tools for Python.

9. Sankoff D, Ferretti V. Karyotype distributions in a stochastic model of reciprocal translocation. Genome Res. 1996;6: 1–9. doi:10.1101/gr.6.1.1

10. Arkendra DE, Ferguson M, Sindi S, Durrett R. The equilibrium distribution for a generalized Sankoff-Ferretti model accurately predicts chromosome size distributions in a wide variety of species. J Appl Probab. 2001;38: 324–334. doi:10.1239/jap/996986747

11. Pevzner P, Tesler G. Human and mouse genomic sequences reveal extensive breakpoint reuse in mammalian evolution. Proc Natl Acad Sci. 2003;100: 7672–7677. doi:10.1073/pnas.1330369100
